# Selective H/D Exchange in E–H (E = Si, Ge, Sn) Bonds Catalyzed by 1,2,3-Triazolylidene-Stabilized Nickel Nanoparticles

**DOI:** 10.1021/acs.inorgchem.5c00216

**Published:** 2025-04-16

**Authors:** Pablo Molinillo, Ana Gálvez Del Postigo, Maxime Puyo, Florencia Vattier, Ana M. Beltrán, Nuria Rendón, Patricia Lara, Andrés Suárez

**Affiliations:** ! Instituto de Investigaciones Químicas (IIQ), Departamento de Química Inorgánica, and Centro de Innovación en Química Avanzada (ORFEO−CINQA), 131736 CSIC and Universidad de Sevilla, Avda. Américo Vespucio, 49, Sevilla 41092, Spain; @ Instituto de Ciencia de Materiales de Sevilla, CSIC-Universidad de Sevilla, Avda. Américo Vespucio 49, Sevilla 41092, Spain; # Departamento de Ingeniería y Ciencia de los Materiales y del Transporte, Escuela Politécnica Superior, Universidad de Sevilla, Sevilla 41011, Spain

## Abstract

Nickel nanoparticles (Ni·MIC) stabilized with mesoionic 1,2,3-triazolylidene (MIC) ligands were prepared via decomposition of the [Ni­(COD)_2_] (COD = 1,5-cyclooctadiene) complex with H_2_ (3 bar) in the presence of 0.2 or 0.5 equiv of ligand. The obtained monodisperse and small-sized (3.2–3.8 nm) nanoparticles were characterized by high-resolution transmission electron microscopy (TEM, HRTEM) and inductively coupled plasma (ICP) analysis. Further analysis of the nickel nanoparticles by X-ray photoelectron spectroscopy (XPS) demonstrated the coordination of the MIC ligands to the metal surface. Finally, the Ni·MIC nanoparticles were applied in the isotopic H/D exchange in hydrides of group 14 elements (Si, Ge, Sn) using D_2_ gas under relatively mild conditions (1.0–1.8 mol % Ni, 1 bar D_2_, 55 °C). High and chemoselective deuterium incorporation at the E–H (E = Si, Ge, Sn) bond in these derivatives was observed.

## Graphical abstract

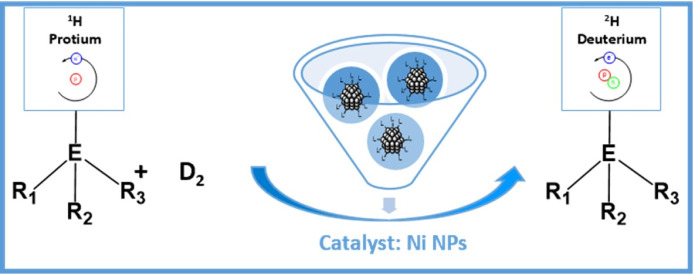

## Introduction

Organic molecules incorporating hydrogen heavy isotopes (i.e., deuterium and tritium) are valuable derivatives that are routinely utilized in drug discovery and development,^1,2^ serve as internal standards in mass spectrometry,^1,3^ and are used in the elucidation of (bio)­chemical reaction mechanisms.^1,4^ These molecules are conveniently accessed through hydrogen isotope exchange (HIE) reactions involving catalytic C–H bond activation, since these methods allow the incorporation of deuterium or tritium at a late stage of the synthetic procedure.^5,6^ In this context, inspired by initial observations of Finke et al.,^7^ several groups particularly those of Chaudret, Rousseau, and Pieters,^8–27^ demonstrated that metal nanoparticles render efficient catalytic systems for H/D exchange in numerous molecules, using readily available and relatively cheap D_2_ gas, due to their ability to activate C–H and H–H bonds under mild conditions.^28^

Hydrosilanes and their heavier group 14 element counterparts are commonly employed as reagents in many organic transformations, such as halogen/hydrogen exchange reactions^29^ and the addition to unsaturated carbon–carbon and carbon–heteroatom bonds (heteroatom = O, N).^30^ Consequently, deuterium-enriched hydrides containing E–D (E = Si, Ge, Sn) bonds can be used in the deuterium labeling of complex organic molecules, as well as in the study of the mechanisms of these reactions. H/D exchange reactions in hydrosilanes using deuterium gas have been reported using transition metal complexes,^31–43^ metal colloids,^44^ and heterogeneous metal catalysts.^45^ While most of these catalytic systems are based on precious metals, first-row transition metal complexes, which have lower economical and environmental costs, have also been recently explored in H/D exchange in Si–H bonds (Figure 1).^38,41–43^ In striking contrast, catalytic systems for H/D exchange in hydrides of silicon’s heavier group 14 elements, such as hydrogermananes and hydrostannanes, which also have important applications in organic synthesis, have been scarcely investigated.^40,44^

**Figure 1:**
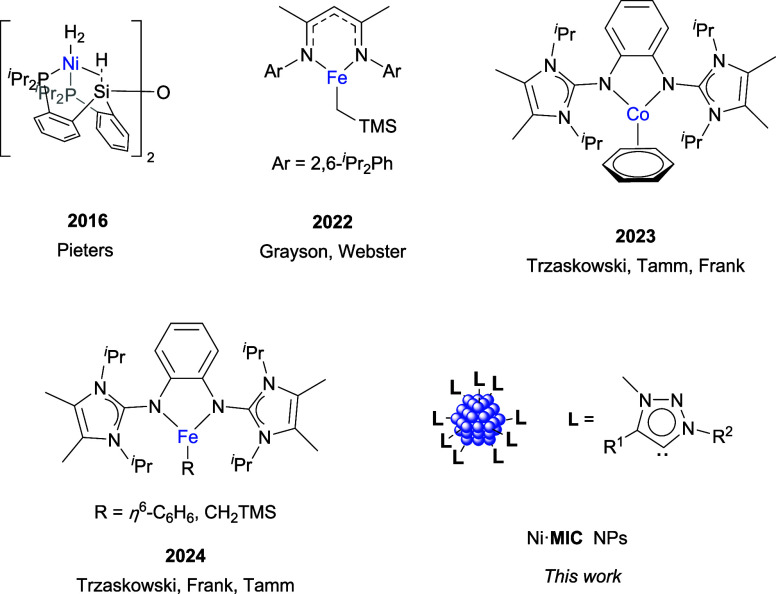
Catalytic systems based on first-row transition metals for H–D exchange in hydrosilanes.

Stabilization of metal nanoparticles by organic ligands allows facile tuning of the size, shape, and surface properties of nanomaterials through easy modification of the structure and amount of the capping ligand.^46^ N-heterocyclic carbenes (NHCs) have become an important class of stabilizing agents in metal nanoparticle preparation due to their easy synthesis, wide structural diversity, and strong electron-donor properties.^47^ While most examples of metal nanoparticles stabilized by NHCs involve the use of commonly employed imidazole-2-ylidene (*u*NHC)^48^ and imidazolidin-2-ylidene (*s*NHC)^49^ ligands, other classes of NHC derivatives, such as 1,2,3-triazolylidenes^50,51^ (MIC) and cyclic (alkyl)­(amino)­carbenes^52^ (CAAC), have received limited attention (Figure 2). Particularly, mesoionic 1,2,3-triazolylidene ligands possess stronger electron-donor properties than more traditional imidazole-2-ylidene (*u*NHC) and imidazolidin-2-ylidene (*s*NHC) derivatives, which makes them appealing stabilizers for metal nanoparticle synthesis.^50^ Recently, several groups have described the synthesis of silver and gold nanoparticles stabilized with MIC ligands.^51^ These nanomaterials were synthesized through the chemical reduction of mixtures of gold salts and MIC ligands generated *in situ* with NaBH_4_ or amine-borane, or using preformed Au- or Ag-MIC complexes. Similarly, our group reported the synthesis of small and monodisperse Ru nanoparticles (1.1–1.8 nm) stabilized by mesoionic 1,2,3-triazolylidene ligands.^44^ The preparation of these nanocatalysts was performed through the decomposition of the ruthenium precursor [Ru­(COD)­(COT)] with H_2_ in the presence of 0.1–0.2 equiv of *in situ* generated MIC ligands. Based on the well-known ability of metal nanoparticles to activate Si–H bonds, these Ru·MIC nanomaterials were examined in the H/D exchange reaction in main-group hydrides having E–H (E = Si, Ge, Sn, and B) bonds, exhibiting good catalytic activity and selectivity under mild reaction conditions (1.0 mol % Ru, 1 bar D_2_, 55 °C, THF).

**Figure 2:**
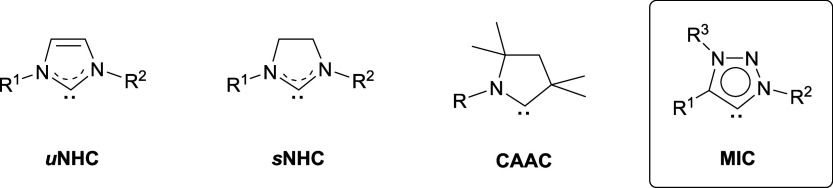
N-heterocyclic carbene ligands used for the stabilization of catalytically active metal nanoparticles.

Herein, we report the extension of our studies on metal nanoparticles incorporating MIC ligands to the synthesis and characterization of small-sized and monodisperse Ni nanoparticles capped with mesoionic 1,2,3-triazolylidene derivatives, as well as their application to the selective deuteration of E–H bonds (E = Si, Ge, Sn) using D_2_ gas. The use of related Ni nanoparticles stabilized with *u*NHCs in the protium-to-deuterium exchange in N-heterocycles has been previously reported by Chaudret et al.^20^

## Results and Discussion

### Synthesis and Characterization of Ni·MIC Nanoparticles

The synthesis of nickel nanoparticles stabilized with 1,2,3-triazolylidene (MIC) ligands was performed by the decomposition of the nickel(0) complex [Ni­(COD)_2_] with H_2_ (3 bar) in the presence of substoichiometric amounts of the corresponding 1,2,3-triazolium tetrafluoroborate salts and potassium bis­(trimethylsilyl)­amide (KHMDS) (MIC/Ni = 0.2 or 0.5 equiv), as previously described in the preparation of Ru·MIC nanoparticles (Scheme 1).^44^ Following this methodology, a series of nanoparticles capped with two different triazolylidene ligands, **MIC1** and **MIC2**, having MIC/Ni ratios of 0.2 and 0.5, was obtained. In all cases, the formation of monodisperse and small nanoparticles (3.2–3.8 nm) was confirmed by TEM analysis (Figure 3, Table 1). As expected, the use of a lower amount of stabilizing ligand (0.2 equiv) resulted in the formation of larger nanoparticles than those obtained with higher amounts of capping molecules (0.5 equiv). In contrast, when the carbene ligand **MIC3** was examined as the capping agent using a MIC/Ni ratio of 0.2, TEM analysis revealed the formation of agglomerates; meanwhile, superstructures composed of individual nanoparticles exhibiting a mean size of 4.1 (0.8) nm were observed upon using **MIC3**/metal ratios of 0.5 (Figure S1 and S2). These results show that **MIC3**, having a linear alkyl substituent at the carbon adjacent to the carbenic carbon, provides limited stabilization of the nanoparticles and points out the positive influence of the presence of a Ph group at the C-5 position of the ligand, as in the case of **MIC1** and **MIC2**, to achieve good nanoparticle stability. Finally, the metal contents of the nanomaterials containing ligands **MIC1** and **MIC2**, which were found to be stable both in the solid state and in solution for weeks under an inert atmosphere, were determined by inductively coupled plasma (ICP) analysis (Table 1).

**Scheme 1:**
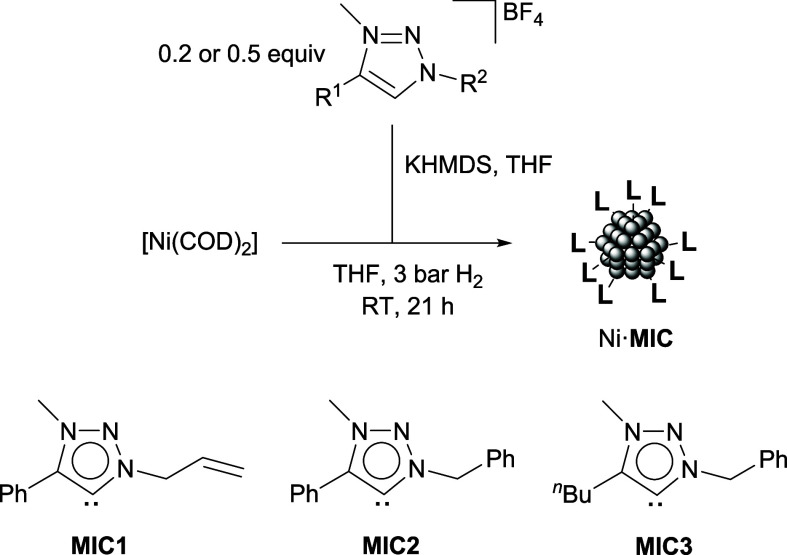
Synthesis of Ni·MIC Nanoparticles and 1,2,3-Triazolylidene (MIC) Ligands Used

**Table 1:** TEM and ICP Analyses of the Ni·MIC Nanoparticles

Ni·MIC	MIC/Ni ratio	%wt Ni^a^	Mean size [nm]^b^
Ni·**MIC1** ^0.2^	0.2	32	3.8 (0.8)
Ni·**MIC2** ^0.2^	0.2	38	3.6 (0.5)
Ni·**MIC1** ^0.5^	0.5	23	3.5 (0.7)
Ni·**MIC2** ^0.5^	0.5	22	3.2 (0.5)

a %wt Ni content as determined by ICP analysis.

b Measured from TEM images. Standard deviations in parentheses.

**Figure 3:**
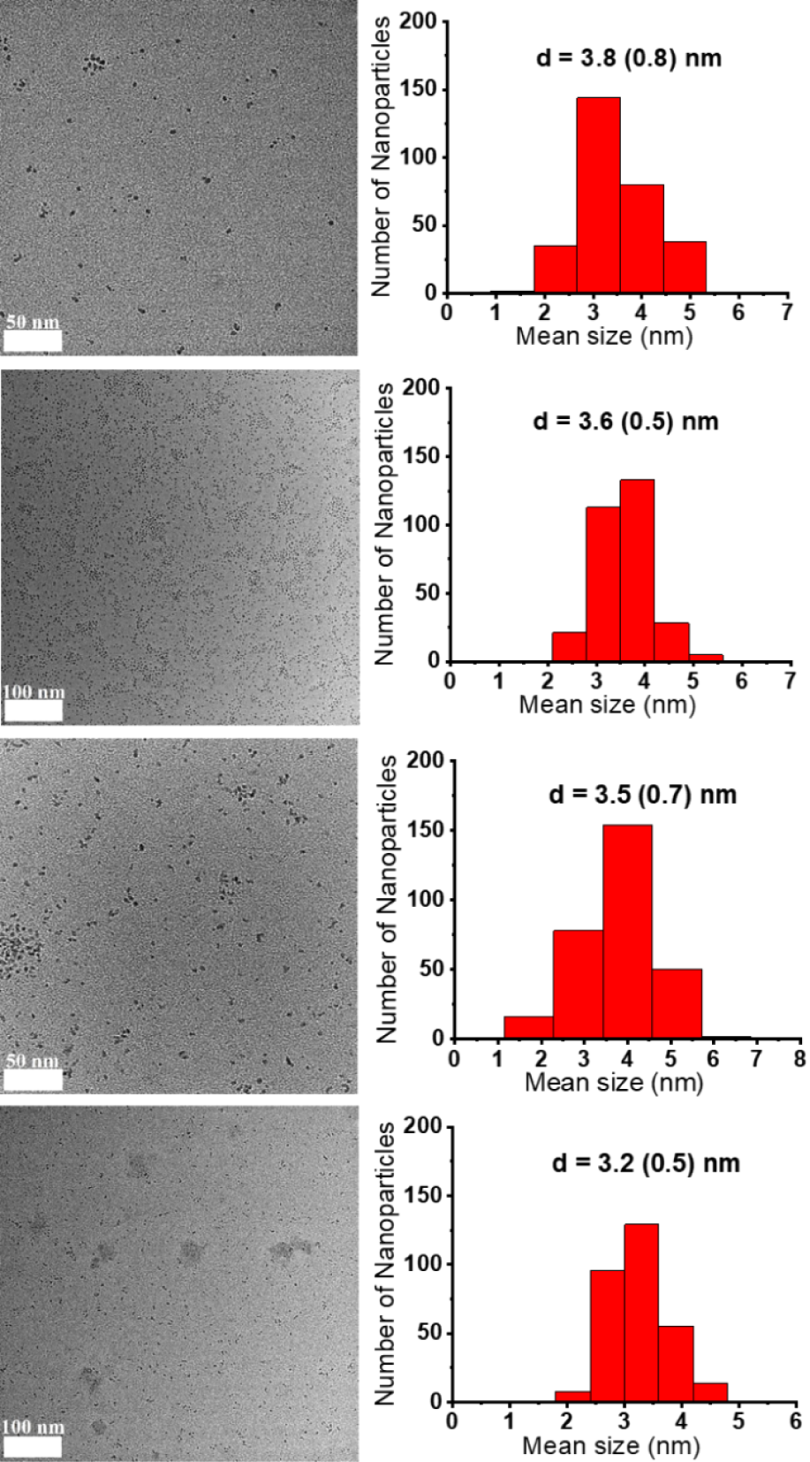
TEM images with size distribution histograms of Ni·**MIC1** ^0.2^, Ni·**MIC2** ^0.2^, Ni·**MIC1** ^0.5^, and Ni·**MIC2** ^0.5^ (from top to bottom).

The crystalline nature of the nanoparticles stabilized by **MIC1** and **MIC2**, which exhibit the expected face-centered cubic (fcc) packing, was confirmed by high-resolution transmission electron microscopy (HRTEM) analysis of the Ni·**MIC1** ^0.2^ and Ni·**MIC2** ^0.2^ nanomaterials as representative examples (Figure 4, top; Figure S3).^20,53^ Moreover, STEM-EDX analysis demonstrates the nickel composition of the nanoparticles, also showing the presence of oxygen peaks, which may have originated from the support material or may be indicative of partial oxidation of the metal particles (Figure 4, bottom; Figure S3).

**Figure 4:**
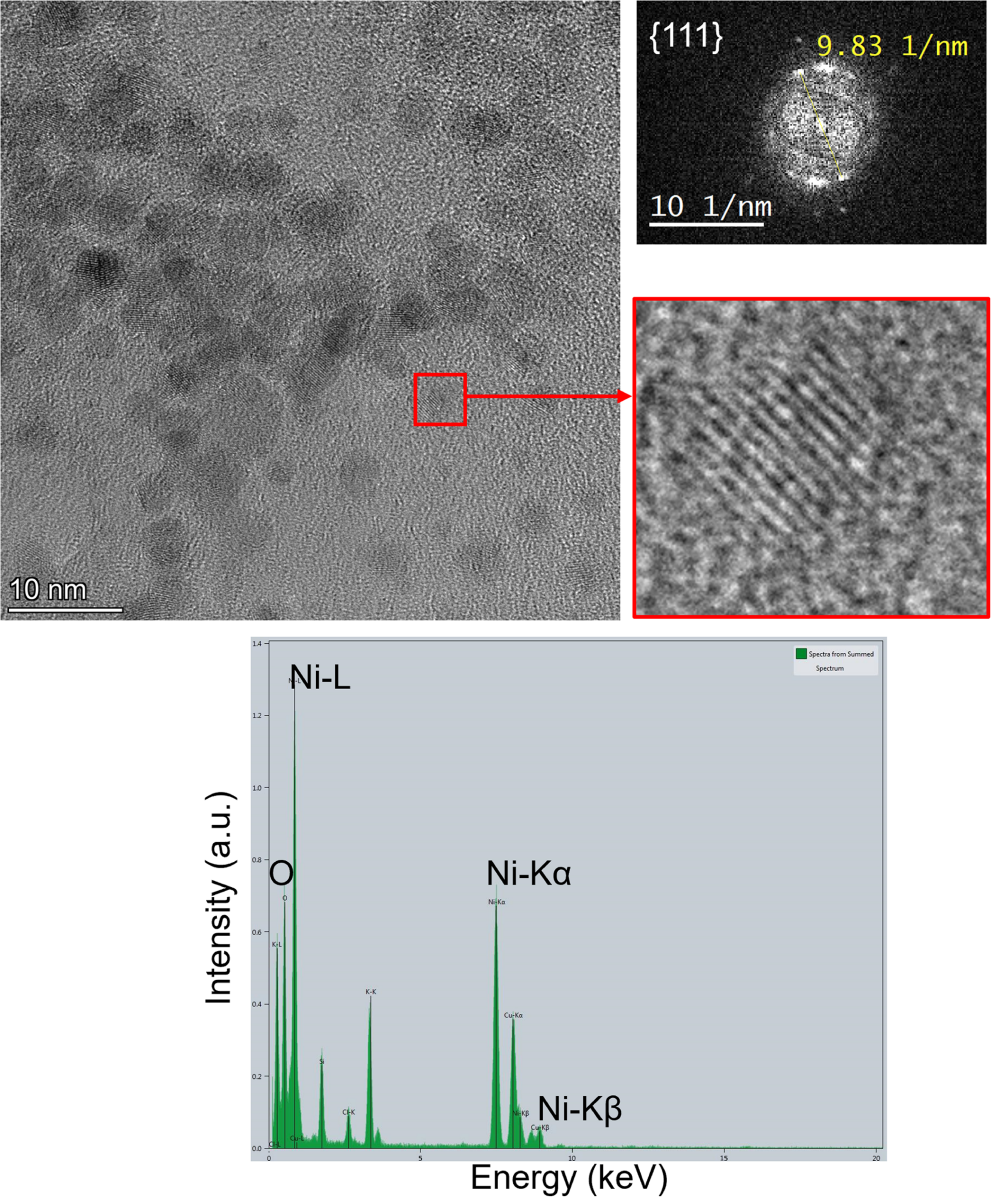
HRTEM images (top) of Ni·**MIC2** ^0.2^ nanoparticles. The corresponding fast Fourier transform analyses of spatial frequencies (right, top) show that the lattice fringes observed on the HRTEM images correspond to planes of the fcc structure. STEM-EDX analysis (bottom) of Ni·**MIC2** ^0.2^ nanoparticles.

Next, X-ray photoelectron spectroscopy (XPS) of the Ni·MIC nanomaterials was used to get information regarding the composition and chemical state of the nanoparticle metal surfaces. In all four samples, the Ni 2p region exhibits two peaks centered at 852.3 and 870.0 eV in binding energy (BE), corresponding to the Ni 2p_3/2_ and 2p_1/2_ photoemission peaks, respectively.^54^ These signals are complex due to the overlap of several contributions from different formal oxidation states and the characteristic satellites of Ni species resulting from Ni(0) and oxidized Ni atoms. To evaluate the thickness of the oxide layer, measurements were conducted at different angles between the sample and the analyzer. As the angle increases from the standard 0° to 45°, the measured layer becomes thinner, making the obtained information more sensitive to the surface. Figure 5 displays the Ni 2p_3/2_ region of the Ni·**MIC1** ^0.2^ nanoparticles. The higher peak for the Ni(0) species appears at 852.3 eV in BE and is accompanied by two satellites due to plasmon loss structures (blue curves).^55^ The overlapping multiplet splitting structures corresponding to NiO (green) and Ni­(OH)_2_ (pink) have their main peaks at 854.1 and 854.8 eV in BE, respectively.^56^ At a lower measurement angle (45 ° take-off), the amount of nickel oxides is higher (Figure 5, bottom). These observations indicate that partial oxidation of the metal is confined to the outer layer of the surface.

**Figure 5:**
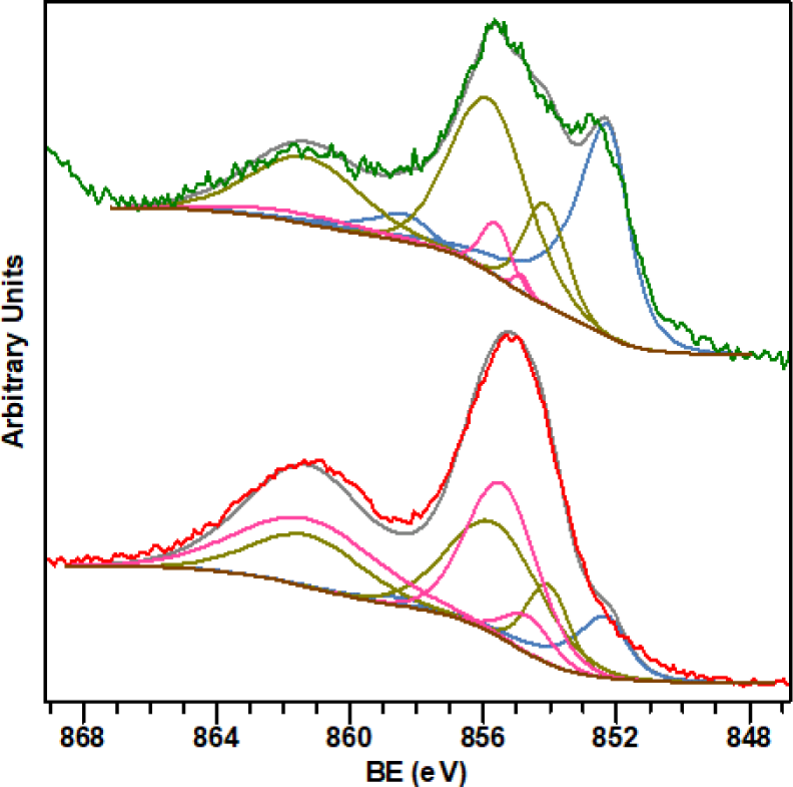
Experimental and fitted spectra of the Ni 2p_3/2_ region for Ni·**MIC1** ^0.2^ measured at 0° (top) and 45° (bottom) take off angles, showing the well fitted multiplet splitting peaks for Ni(0) (blue line), NiO (green line), and Ni­(OH)_2_ (pink line) species.

Moreover, the outer hydroxide and oxide layer was easily removed by ionized Ar sputtering under mild conditions, indicating that the layer was quite thin. The treatment was conducted while ensuring that the N 1s signal of the carbene ligand remained unchanged. Figure 6 shows the high-resolution photoemission Ni 2p_3/2_ region for a freshly prepared Ni·**MIC1** ^0.2^ sample (top) and after cleaning by Ar^+^ sputtering (bottom), along with the fitting spectra for Ni(0) (blue line) and oxidized Ni species (NiO, green line; Ni­(OH)_2_, orange line). Ni(0) accounts for 35% of the atomic concentration on the surface of the original nanoparticles, increasing to 60% after sputtering. Consequently, it seems reasonable to assume that the nanoparticle surface state after the mild etching treatment is comparable to that of the nanoparticles synthesized under an inert atmosphere, and therefore the predominant oxidation state of the Ni atoms on the nanoparticle surface is 0, although partial oxidation occurs during the sample preparation and introduction into the XPS equipment. Similar results were obtained in the analysis of the XPS spectra for the samples Ni·**MIC1** ^0.5^, Ni·**MIC2** ^0.2^, and Ni·**MIC2** ^0.5^ (Figure S5).

**Figure 6:**
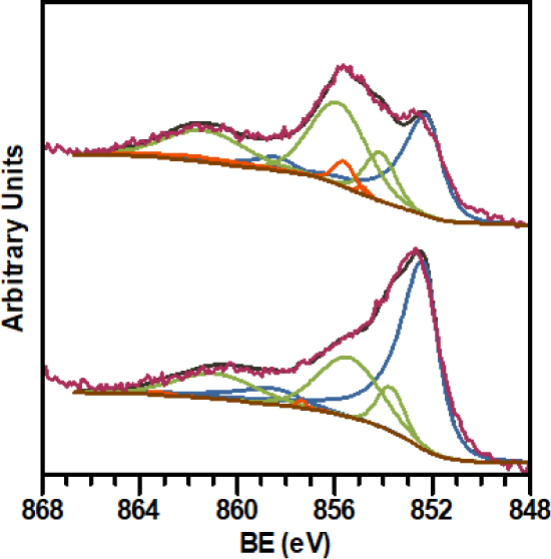
Experimental and fitted XPS spectra of the Ni 2p_3/2_ region for Ni·**MIC1** ^0.2^ nanoparticles freshly prepared (top) and cleaned by Ar^+^ sputtering (bottom). Ni(0) (blue), NiO (green), and Ni­(OH)_2_ (orange).

X-ray photoelectron spectroscopy was also employed to determine the presence of the coordinated MIC ligands on the surfaces of the Ni·MIC nanoparticles. Figure 7 shows the high-resolution spectra of the N 1s region of Ni·**MIC1** ^0.2^ and Ni·**MIC2** ^0.2^ nanoparticles. The N 1s photoemission peak of the Ni·**MIC1** ^0.2^ sample (Figure 7; left, bottom) consists of a signal centered at 399 eV, which can be deconvoluted into two components at 400.3 and 398.6 eV, with a peak area ratio of 2:1 (fwhm = 2.5–3 eV).^57^ The lowest BE peak at 398.6 eV can be assigned to the N atom adjacent to the carbene C atom, whereas the peak appearing at higher BE, 400.3 eV, can be attributed to the two N atoms located more distal to the carbenic carbon atom. Coordination of the MIC ligand to the Ni surface through the carbenic carbon should give rise to a peak appearing at a higher BE value than that of the free carbene ligand, due to the loss of charge density on the N atom adjacent to the donating carbon atom.^44,51^ Since **MIC1** and **MIC2** are not isolable carbenes, the triazolium salt precursors of **MIC1** and **MIC2** were also analyzed by XPS in lieu of the free ligands. The N 1s signal for the Ni·**MIC1** ^0.2^ nanoparticles appears as a broadened peak at lower BE than that observed for the same peak of the triazolium salt precursor of **MIC1**. This behavior is in line with previous results of metal nanoparticles having a direct coordination of the NHC ligands to the metal surfaces, including related Ru·MIC nanomaterials stabilized by the **MIC1** and **MIC2** ligands.^44^ Similar results were obtained for the Ni·**MIC2** ^0.2^ nanomaterial.

**Figure 7:**
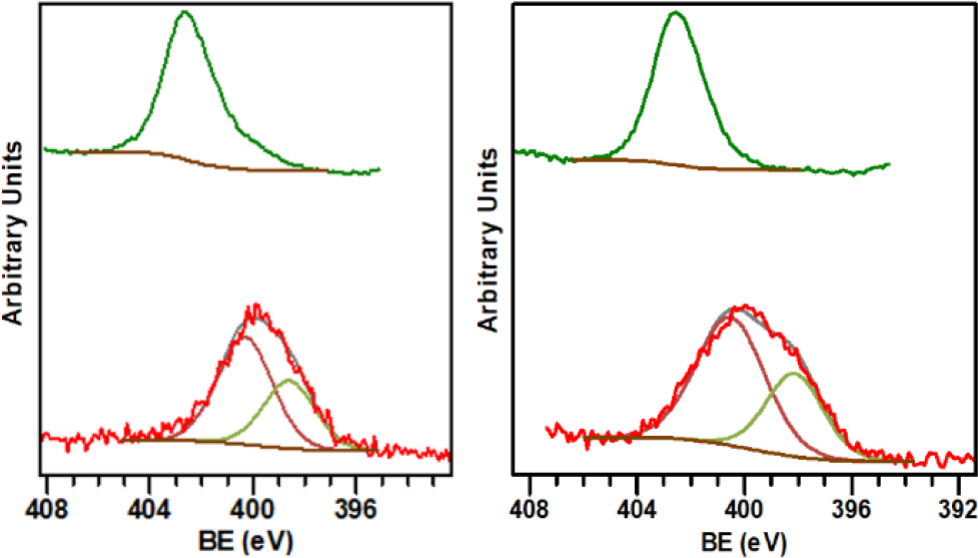
High resolution XPS spectra of the N 1s region for Ni·**MIC1** ^0.2^ (bottom, left) and Ni·**MIC2** ^0.2^ (bottom, right). N 1s region of the triazolium salt precursors of **MIC1** (top, left) and **MIC2** (top, right) ligands are also shown.

Finally, Table 2 presents the atomic concentration percentages of N and Ni along with the Ni/N ratio for each characterized nickel sample. As anticipated, Ni nanoparticles prepared using 0.5 equiv of stabilizing ligand exhibit a higher degree of surface coating than those having a MIC/Ni ratio of 0.2. On the other hand, the larger size of the substituent on the N atom adjacent to the carbenic carbon in **MIC2** makes its accommodation on the surface more difficult due to its higher steric hindrance compared to **MIC1**, which is in agreement with the higher Ni/N ratio obtained for the Ni·**MIC2** nanoparticles.

**Table 2:** Quantitative Analysis of the Surface Composition of Ni·MIC Nanoparticles (Percentage in Atomic Concentration, %at)

Ni·MIC	Ni (%at)	N (%at)	Ni/N ratio
Ni·**MIC1** ^0.2^	49	51	0.9
Ni·**MIC2** ^0.2^	64	36	1.8
Ni·**MIC1** ^0.5^	41	59	0.7
Ni·**MIC2** ^0.5^	60	40	1.5

### Catalytic H/D Exchange

In order to compare the catalytic performance of the various synthesized Ni·MIC nanoparticles, H/D exchange in PhMe_2_SiH (**Si-1**) using D_2_ gas (1 bar, ca. 1.4 equiv) as a deuterium source was examined in THF at 55 °C (Table 3). All the Ni·MIC nanoparticles exhibited good catalytic activity in the reaction, selectively performing the protium-to-deuterium exchange at the SiH position with enrichments higher than 80% (entries 1–4). Among the catalysts in the series, Ni·**MIC1** ^0.2^ nanoparticles provided the highest level of deuterium incorporation. Analysis using TEM of the Ni·**MIC1** ^0.2^ nanoparticles after the catalytic reaction of **Si-1** showed that the size of the particles was maintained (mean size: 3.7 (0.7) nm; Figure S4), demonstrating postcatalysis nanomaterial integrity. Moreover, when the deuteration reaction of **Si-1** was carried out using Ni·**MIC1** ^0.2^ nanoparticles exposed to air for 5 days, significant silane decomposition was observed (Figure S25). This suggests that the oxidation state of the metal at the surface of the particles is critical for achieving chemoselective H/D exchange in the silanes and that the predominant oxidation state of Ni in the freshly prepared catalyst is zero.

**Table 3:** H/D Exchange in PhMe_2_SiH (**Si-1**) Using Ni-MIC Nanoparticles^a^

Entry	Ni·MIC	D incorporation (%)
1	Ni·**MIC1** ^0.2^	87
2	Ni·**MIC2** ^0.2^	80
3	Ni·**MIC1** ^0.5^	80
4	Ni·**MIC2** ^0.5^	85

a Reaction conditions: 1.0 mol % Ni, 1 bar D_2_ (ca. 1.4 equiv D_2_), 55 °C, THF. [S] = 1.3 M. Reaction time: 21 h. A single D_2_ loading was used. Deuterium incorporation as determined by ^1^H NMR spectroscopy. H/D exchange selectivity determined by ^2^H NMR spectroscopy.

Based on these results, the Ni·**MIC1** ^0.2^ nanocatalyst was further examined in the H/D exchange of other silanes (Table 4). Diphenylmethyl- and tripropylsilane, **Si-2** and **Si-3**, were deuterated with high isotopic enrichment, while the conversion of triphenylsilane (**Si-4**) was moderate (entries 1–3). Similarly, 1,2-(Me_2_SiH)­C_6_H_4_ (**Si-5**), a bis­(hydrosilane), exhibited a conversion of 80% to the corresponding deuterated isotopologue under the same reaction conditions (entry 4). Finally, deuterium labeling of triethoxysilane (**Si-6**), an alkoxysilane, was accomplished selectively with high deuterium incorporation (93%; entry 5).

**Table 4:** H/D Exchange in Hydrides of Group 14 Elements Catalyzed by Ni·**MIC1** ^0.2^ ^a^

Entry	E–H	D incorporation (%)
1	Ph_2_MeSiH (**Si-2**)	92
2	*^n^* Pr_3_SiH (**Si-3**)	>99
3	Ph_3_SiH (**Si-4**)	63
4	1,2-(Me_2_SiH)C_6_H_4_ (**Si-5**)	80
5	(EtO)_3_SiH (**Si-6**)	93
6	Et_3_GeH (**Ge-1**)	65
7^b^	Ph_3_GeH (**Ge-2**)	0
8^b^	*^n^* Bu_3_SnH (**Sn-1**)	>99

a Reaction conditions, unless otherwise noted: 1.8 mol % Ni, 1 bar D_2_ (ca. 2.2 equiv D_2_), 55 °C, THF. [S] = 0.8 M. Reaction time: 21 h. A single D_2_ loading was used. Deuterium incorporation as determined by ^1^H NMR spectroscopy. H/D exchange selectivity determined by ^2^H NMR spectroscopy.

b In the absence of light.

Since Chaudret et al. demonstrated that Ni nanoparticles stabilized by imidazole-2-ylidene ligands were active in the deuteration of N-heterocycles, we tested the Ni·**MIC2** ^0.2^ colloids in the H/D exchange in 2-phenylpyridine as a model substrate. Under analogous conditions to those employed by Chaudret et al. (10 mol % Ni, 2 bar D_2_) for the deuteration of N-heterocycles, H/D exchange of the substrate was not observed (Figure S22). Moreover, when a 1:1 mixture of **Si-1** and 2-phenylpyridine in THF was exposed to D_2_ (1 bar) in the presence of the Ni·**MIC1** ^0.2^ nanoparticles (1.8 mol % Ni), selective deuteration of the Si–H moiety was achieved, providing further support for the preferred H/D exchange in Si–H over C–H bonds (Figures S23 and S24).

In addition to silanes, selected examples of germanane (**Ge-1** and **Ge-2**) and stannane (**Sn-1**) derivatives were submitted to H/D exchange with Ni·**MIC1** ^0.2^ nanoparticles (entries 6–8). While an excellent performance was maintained for the stannane **Sn-1** (>99%), this was not the case for germananes, as exemplified by **Ge-1** and **Ge-2**, which were deuterated with moderate enrichment (65%) or no conversion, respectively.

## Conclusions

A series of nickel nanoparticles stabilized by 1,2,3-triazolylidene (MIC) ligands exhibiting small particle size and narrow size distributions has been prepared and characterized by TEM, HRTEM, XPS, and ICP analysis. The resulting nanomaterials efficiently catalyze the selective H/D exchange in E–H (E = Si, Ge, or Sn) bonds using D_2_ gas under relatively mild conditions. To the best of our knowledge, these Ni·MIC nanoparticles represent the first example of first-row transition metal nanocatalysts that are active in the hydrogen isotope labeling of synthetically important silanes, and the only example of base metal catalysts for the deuteration of germananes and stannanes.

## Experimental Procedures

### General Procedures and Nanoparticles Characterization Techniques

All reactions and manipulations were performed under nitrogen or argon, either in an MBraun Unilab Pro glovebox or using standard Schlenk-type techniques. Solvents were distilled under nitrogen with the following desiccants: sodium benzophenone ketyl for tetrahydrofuran (THF) and sodium for pentane. [Ni­(COD)_2_] (98%) and D_2_ (99.8% D) were purchased from Strem Chemicals and Merck, respectively. 1,2,3-Triazolylidene ligand precursors were synthesized using previously reported procedures.^44^ All of the other chemicals were used as received from commercial suppliers.

The morphology and size of the 1,2,3-triazolylidene-stabilized nickel nanoparticles (Ni·MIC) were determined by transmission electron microscopy (TEM) on an FEI TALOS F200S apparatus operating at 200 kV at the Centro de Investigación, Tecnología e Innovación-CITIUS (Universidad de Sevilla). TEM samples were prepared by taking a drop of the crude THF colloidal solution, depositing it over a covered holey copper grid, and analyzing it under atmospheric conditions. For the approximation of the particles’ mean size, ca. 300 particles were manually measured employing conventional TEM micrographs enlarged with ImageJ software. High-resolution transmission electron microscopy (HRTEM) images to assess the crystal structure of the Ni·**MIC1** ^0.2^ and Ni·**MIC2** ^0.2^ nanoparticles were recorded on a Thermo Scientific Talos F200X microscope under a 200 kV-accelerated electron beam, with a 40-μm objective aperture, ensuring that one first-neighbor reflections, in the Bragg diffraction sense, contributed to the HRTEM micrographs. Scanning transmission electron microscopy with energy dispersive X-ray spectroscopy (STEM-EDX) images using scanning TEM mode combined with energy-dispersive X-ray spectrometry were acquired over various nanoparticles to assess their composition. A small electron probe (size ca. 0.5 nm, current 500 pA) was scanned over an area of 140 × 160 pixels with a dwell time of 50 μs/pixel, and the EDX signal was integrated over about 200 frames using drift correction. The high-angle annular dark-field (HAADF) signal was also recorded simultaneously to locate the nanoparticles. Net integrated intensity maps of the Ni-L line were then extracted after applying a set of pre- and postfiltering functions using the Velox software.

ICP analyses were performed at Mikroanalytisches Labor Pascher (Remagen, Germany).

X-ray photoelectron spectroscopy (XPS) experiments were performed in a PHOIBOS-100 spectrometer with nonmonochromatic Mg–Kα radiation (*h*ν = 1235.6 eV) and a power source of 230 W. Samples were prepared in a glovebox and brought to the spectrometer under an inert atmosphere to reduce nanoparticle exposure to air. However, samples’ insertion into the spectrometer was carried out under atmospheric conditions. The electron energy hemispherical analyzer was operated in constant pass energy mode (SPECS PHOIBOS 100DLD). Low-resolution survey spectra were obtained with a pass energy of 50 eV, while high-energy resolution spectra of detected elements were obtained with a pass energy of 30 eV. The spectra were analyzed with the CASA XPS software, version 2.3.16.Dev52 (Neal Fairly, UK). Shirley-type backgrounds were used to determine the areas under the peaks.

### Nickel Nanoparticles Synthesis

[Ni­(COD)_2_] (0.250 g, 0.90 mmol), 1,2,3-triazolylidene ligand precursor (0.18 mmol for Ni·**MIC1** ^0.2^ and Ni·**MIC2** ^0.2^ nanoparticles; 0.45 mmol in the case of Ni·**MIC1** ^0.5^ and Ni·**MIC2** ^0.5^), and KHMDS (0.043 g, 0.18 mmol for Ni·**MIC1** ^0.2^ and Ni·**MIC2** ^0.2^ nanoparticles; 0.100 g, 0.45 mmol in the case of Ni·**MIC1** ^0.5^ and Ni·**MIC2** ^0.5^) were introduced into a Fisher-Porter vessel and cooled to −50 °C. To the mixture, 200 mL of freshly distilled and degassed by N_2_ bubbling THF was added. The Fisher-Porter vessel was pressurized with 3 bar of H_2_, and the solution was left to slowly reach room temperature under vigorous stirring. The homogeneous solution was kept under stirring overnight, and the H_2_ pressure was carefully released. The initial volume of solvent was reduced to ca. 5 mL under reduced pressure, 30 mL of pentane was added, and the colloidal suspension was stirred for 5 min; volatiles were removed under vacuum. Ni·MIC nanoparticles were obtained as black solids after extended drying. For Ni content determination by ICP and TEM analysis of the nanoparticles, see Table 1.

### Representative Procedure for the Selective H/D Exchange of Dimethylphenylsilane (**Si-1**) (Table 3)

In a glovebox, a 25 mL Fisher-Porter vessel was charged with a solution of dimethylphenylsilane (**Si-1**) (209 μL, 1.35 mmol) and Ni·**MIC1** ^0.2^ (2.0 mg, 13 μmol) in THF (1.0 mL). The reactor was purged three times with D_2_, and finally pressurized to 1 bar and heated to 55 °C. After 21 h, the reactor was slowly cooled down to room temperature and depressurized. An aliquot of the reaction mixture was filtered through a short pad of Celite and brought to dryness. The conversion and selectivity were determined by ^1^H and ^2^H NMR spectroscopies, respectively.

### Representative Procedure for the Selective H/D Exchange of E–H (E = Si, Ge, Sn) Bonds (Table 4)

In a glovebox, a 25 mL Fisher-Porter vessel was charged with a solution of triphenylsilane (**Si-4**) (180 μL, 0.77 mmol) and Ni·**MIC1** ^0.2^ (2.0 mg, 13 μmol) in THF (1.0 mL). The reactor was purged three times with D_2_, and finally pressurized to 1 bar and heated to 55 °C. After 21 h, the reactor was slowly cooled down to room temperature and depressurized. An aliquot of the reaction mixture was filtered through a short pad of Celite and brought to dryness. The conversion and selectivity were determined by ^1^H and ^2^H NMR spectroscopies, respectively.

No uncommon hazards were noted.

## Supplementary Material



## Data Availability

Data will be made available on request.

## Notes

TEM images of Ni·**MIC3** nanomaterials; HRTEM images, fast Fourier transform analyses of spatial frequencies and intensity maps of Ni·**MIC1** ^0.2^; postcatalysis TEM images and size distribution histogram of Ni·**MIC1** ^0.2^; XPS spectra of Ni·**MIC1** ^0.5^, Ni·**MIC2** ^0.2^, and Ni·**MIC2** ^0.5^; NMR data of deuterated compounds (PDF)
