# Supplementary material for: Selective H/D Exchange in E–H (E = Si, Ge, Sn) Bonds Catalyzed by 1,2,3-Triazolylidene-Stabilized Nickel Nanoparticles
Source: Inorg Chem. 2025 Apr 16;64(16):8125–34. doi: 10.1021/acs.inorgchem.5c00216 (PMC12135044; doi:10.1021/acs.inorgchem.5c00216)
Supplement: Supplementary file 1 [file ic5c00216_si_001.pdf]

Supporting Information

Selective H/D Exchange in E-H (E = Si, Ge, Sn) Bonds Catalyzed by 1,2,3-Triazolylidene-Stabilized Nickel Nanoparticles

Pablo Molinillo,[a] Ana Gálvez del Postigo,[a] Maxime Puyo,[a] Florencia Vattier,[b] Ana M. Beltrán,[c] Nuria Rendón,*[a] Patricia Lara*[a] and Andrés Suárez*[a]

[a] *Instituto de Investigaciones Químicas (IIQ), Departamento de Química Inorgánica, and Centro de Innovación en Química Avanzada (ORFEO-CINQA). CSIC and Universidad de Sevilla. Avda. Américo Vespucio, 49, 41092 Sevilla, Spain.*

[b] *Instituto de Ciencia de Materiales de Sevilla. CSIC-Universidad de Sevilla. Avda. Américo Vespucio 49, 41092 Sevilla, Spain.*

[c] *Departamento de Ingeniería y Ciencia de los Materiales y del Transporte, Escuela Politécnica Superior, Universidad de Sevilla, 41011 Sevilla, Spain.*

E-mail: nuria@iiq.csic.es; patricia@iiq.csic.es; andres.suarez@iiq.csic.es

Table of contents:

S1. General procedures, materials and characterization techniques.

S2. TEM and HRTEM images.

S3. XPS spectra.

S4. NMR data of deuterated products.

S5. Additional catalytic experiments.

S6. References.

S1. General procedures, materials and characterization techniques.

Liquid phase ^{1}H and ^{2}H NMR spectra were recorded on a Bruker DRX-400 spectrometer. Spectra were referenced to $SiMe_4$ (δ = 0 ppm) using the residual proton solvent peaks as internal standards. All NMR spectra were recorded at 25 ^{o}C.

S2. TEM and HRTEM images.

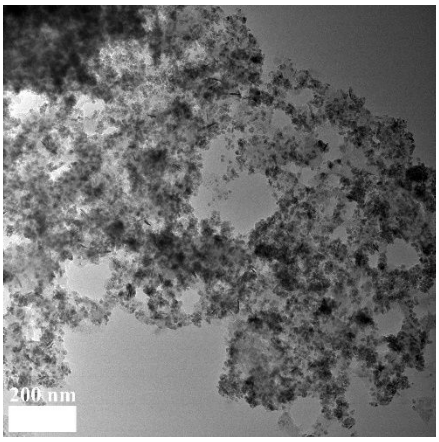

Figure S1. TEM image of the attempted synthesis of Ni·**MIC3**$^{0.2}$ nanoparticles.

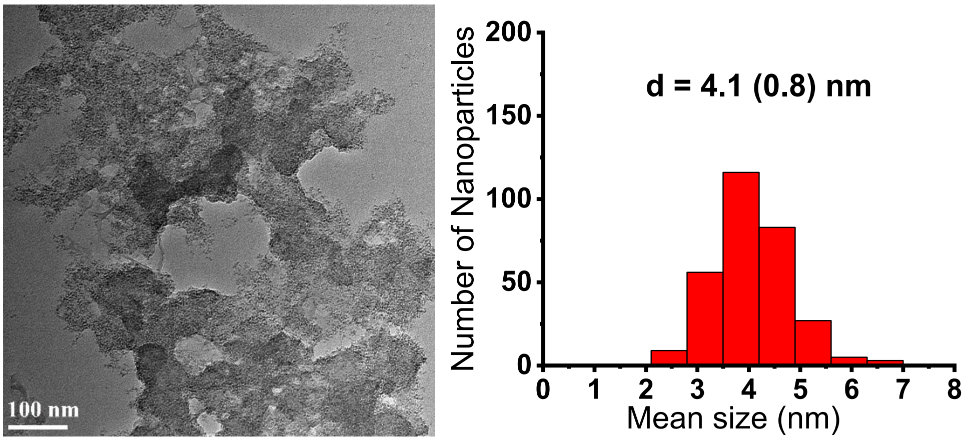

Figure S2. TEM image with size distribution histogram of Ni·**MIC3**$^{0.5}$ nanoparticles.

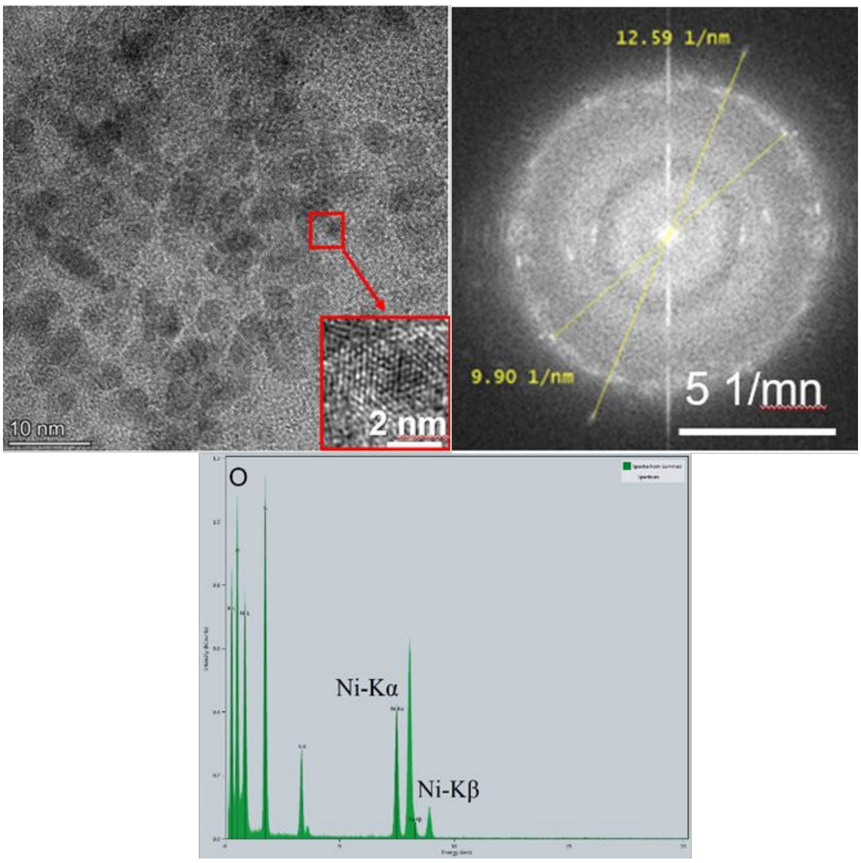

Figure S3. HRTEM images (top, left) of the Ni·**MIC1**$^{0.2}$ nanoparticles and the corresponding Fast Fourier Transform analyses of spatial frequencies (top, right). STEM-EDX analysis (bottom) of the Ni·**MIC1**$^{0.2}$.

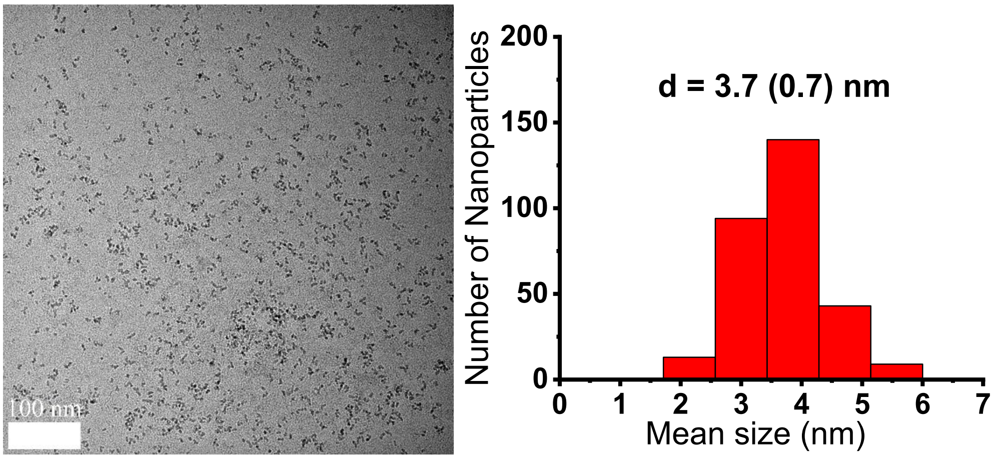

Figure S4. TEM images and size distribution histogram of Ni·**MIC1**$^{0.2}$ after deuteration of **Si-1**.

S3. XPS spectra.

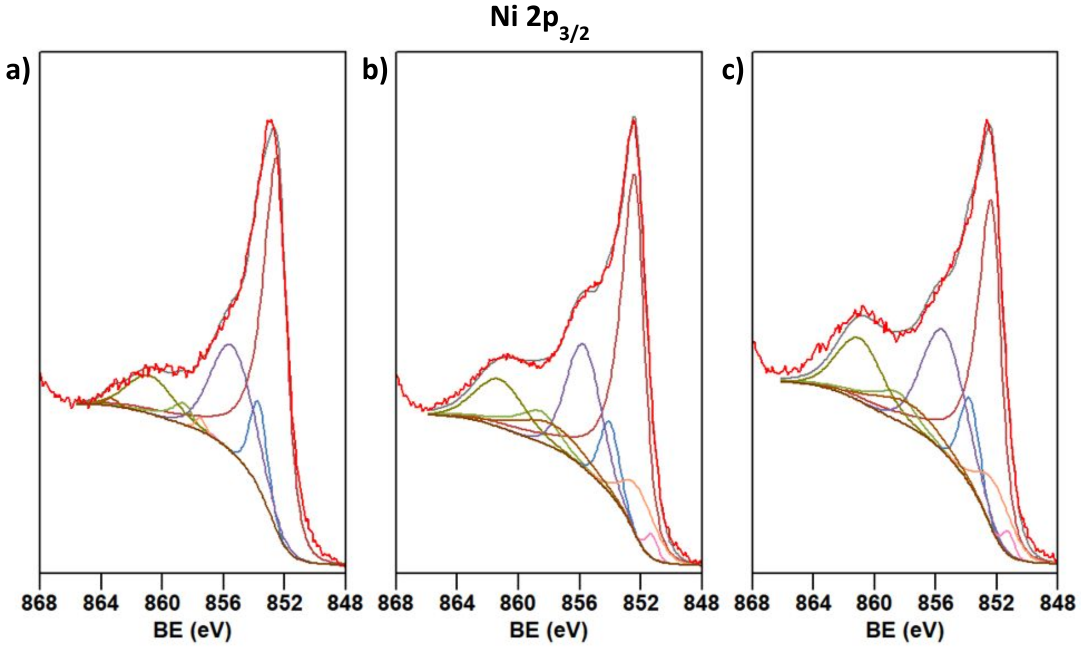

Figure S5. Experimental and fitted high resolution XPS spectra of Ni $2p_{3/2}$ photoemission region for a) Ni·**MIC1**$^{0.5}$, b) Ni·**MIC2**$^{0.2}$, and c) Ni·**MIC2**$^{0.5}$. Ni(0) (blue), NiO (green) and $Ni(OH)_2$ (orange).

S4. NMR data of deuterated products.

Me_2PhSiD (Si-1)

Deuterium incorporation: 87%. Spectroscopic data for this product agrees to those previously reported in the literature.[1,2]

^{1}H NMR (400 MHz, CD_2Cl_2): δ 7.60 (m, 2H, 2 H arom), 7.42 (m, 3H, 3 H arom), 4.48 (hept, $^3J_{HH}$ = 3.8 Hz, SiH), 0.40 (s, 6H, 2 CH_3).

^{2}H NMR (60 MHz, CH_2Cl_2): δ 4.61 (SiD).

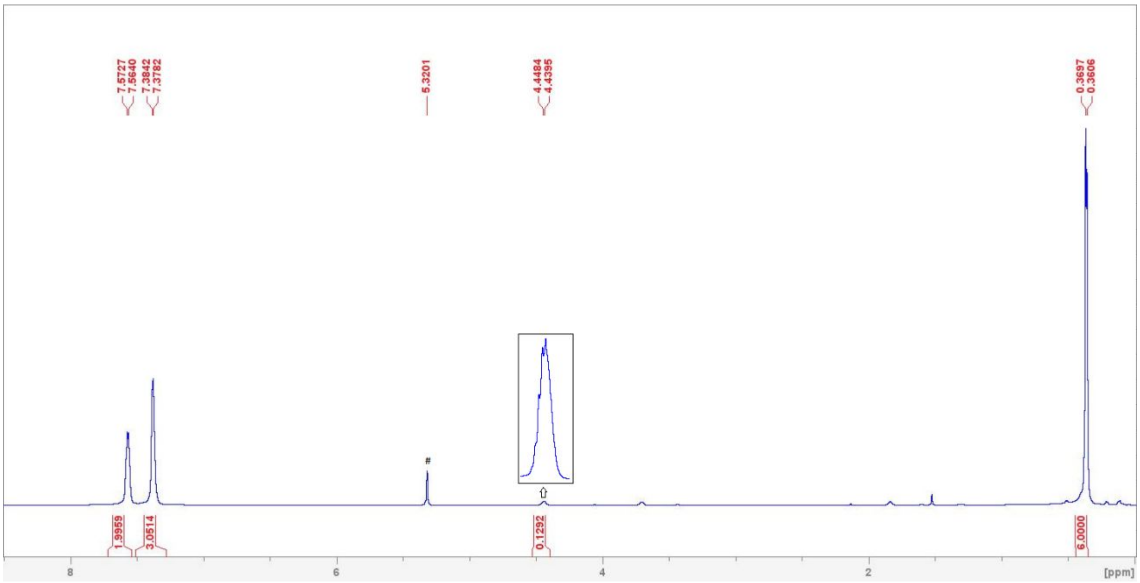

Figure S6. ^{1}H NMR spectrum (CD_2Cl_2, 400 MHz) of the H/D exchange in Me_2PhSiH (**Si-1**). (# denotes residual CH_2Cl_2 from the deuterated solvent).

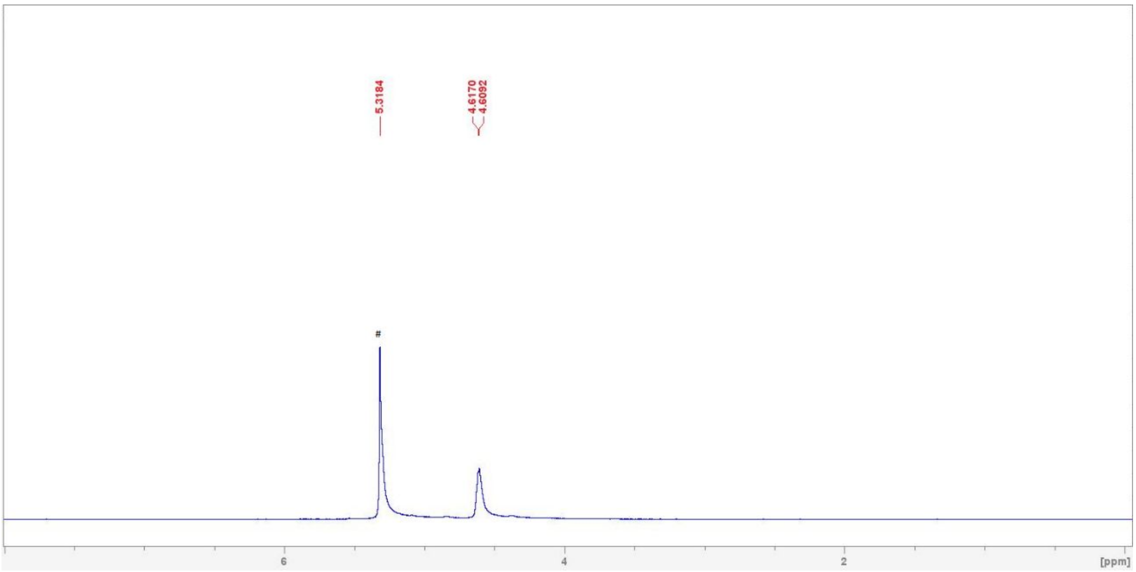

Figure S7. ^{2}H NMR spectrum (CH_2Cl_2, 60 MHz) of the H/D exchange in Me_2PhSiH (**Si-1**). (# denotes CD_2Cl_2 used as reference).

$MePh_2SiD$ (Si-2)

Deuterium incorporation: 92%. Spectroscopic data for this product agree to those previously reported in the literature.[1,3]

^{1}H NMR (400 MHz, CD_2Cl_2): δ 7.62 (m, 4H, 4 H arom), 7.42 (m, 6H, 6 H arom), 4.99 (q, $^3J_{HH}$ = 3.8 Hz, SiH), 0.68 (s, 3H, CH_3).

^{2}H NMR (60 MHz, CH_2Cl_2): δ 5.06 (SiD).

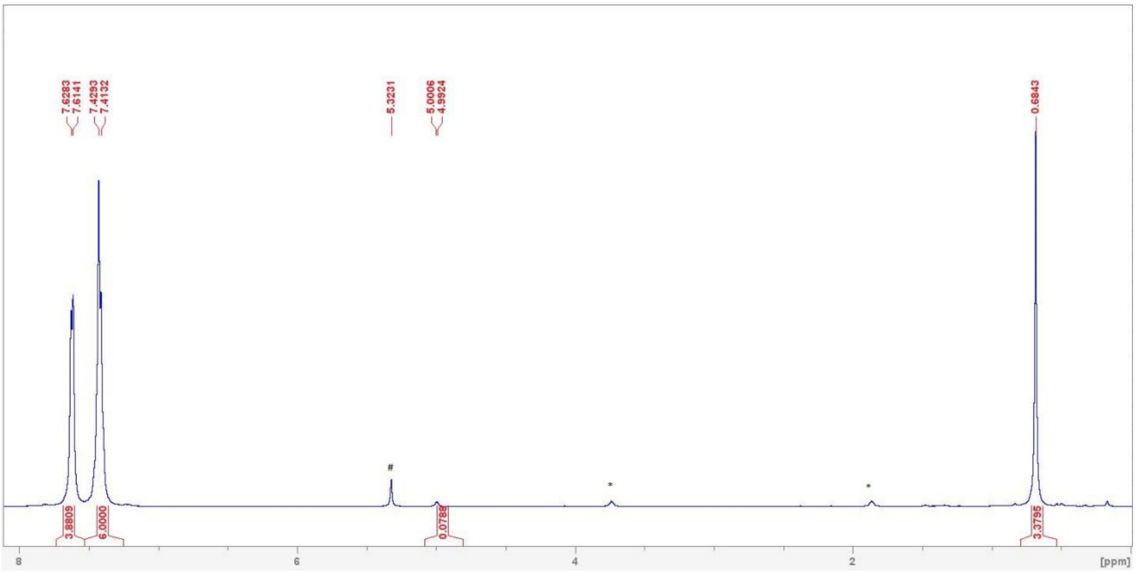

Figure S8. ^{1}H NMR spectrum (CD_2Cl_2, 400 MHz) of the H/D exchange in $MePh_2SiH$ (**Si-2**). (# denotes residual CH_2Cl_2 from the deuterated solvent, * denotes residual THF).

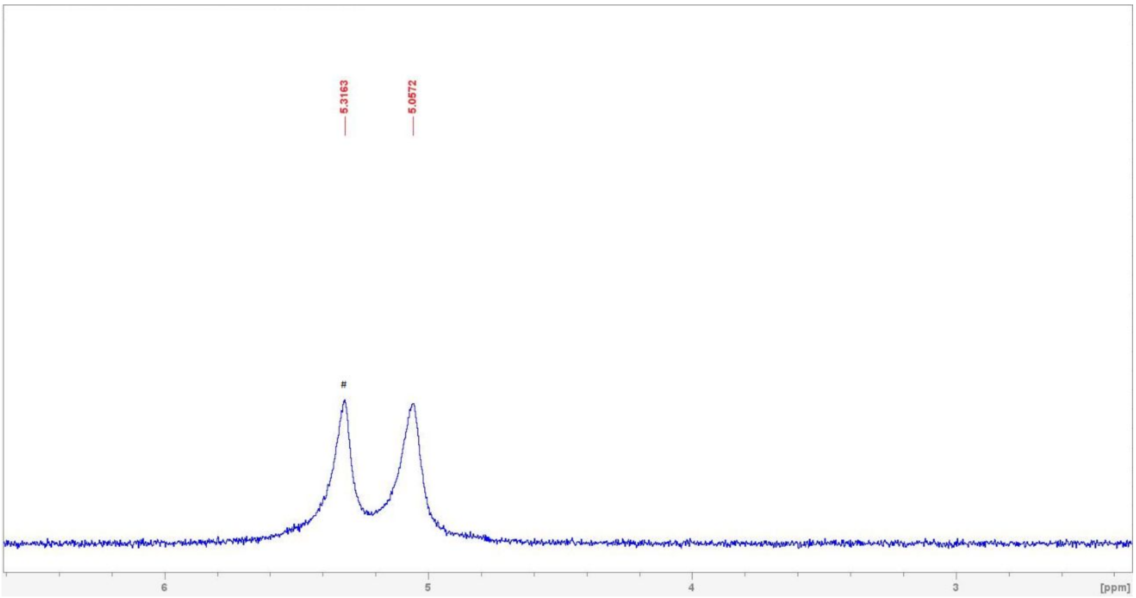

Figure S9. ^2H NMR spectrum (CH_2Cl_2, 60 MHz) of the H/D exchange in $MePh_2SiH$ (**Si-2**). (# denotes CD_2Cl_2 used as reference).

$^n Pr_3SiD$ (Si-3)

Deuterium incorporation: >99%. Spectroscopic data for this product agree to those previously reported in the literature.[1,4]

^1H NMR (400 MHz, CD_2Cl_2): δ 1.35 (m, $^3J_{HH}$ = 7.2 Hz, 6H, 3 CH_2), 0.92 (t, $^3J_{HH}$ = 7.2 Hz, 9H, 3 CH_3), 0.56 (t, $^3J_{HH}$ = 8.1 Hz, 6H, 3 $SiCH_2$).

^2H NMR (60 MHz, CH_2Cl_2): δ 3.70 (SiD).

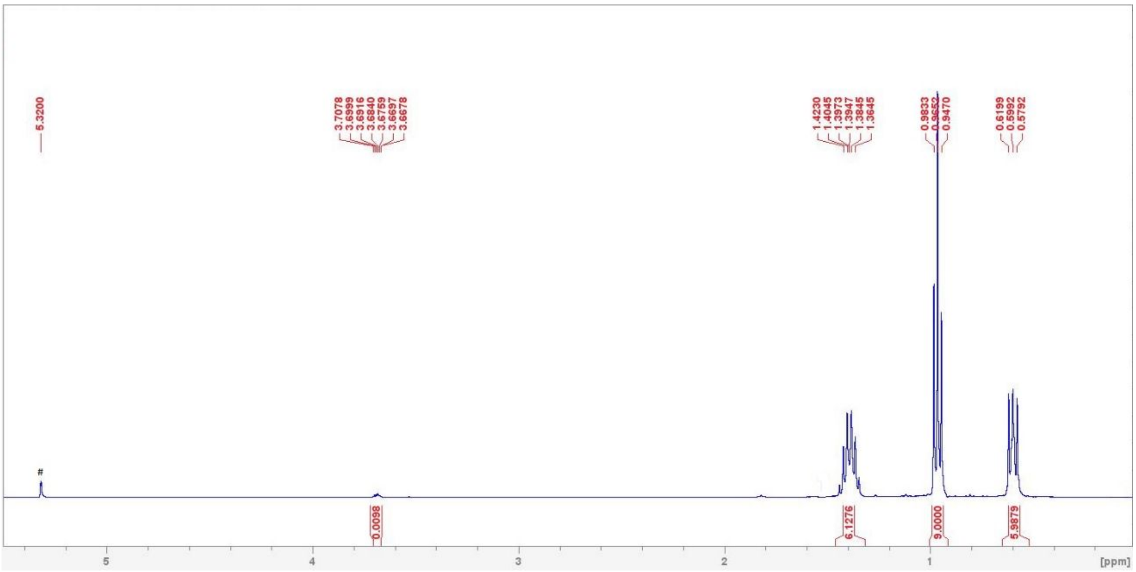

Figure S10. ^1H NMR spectrum (CD_2Cl_2, 400 MHz) of the H/D exchange in $^nPr_3SiH$ (**Si-3**). (# denotes residual CH_2Cl_2 from the deuterated solvent).

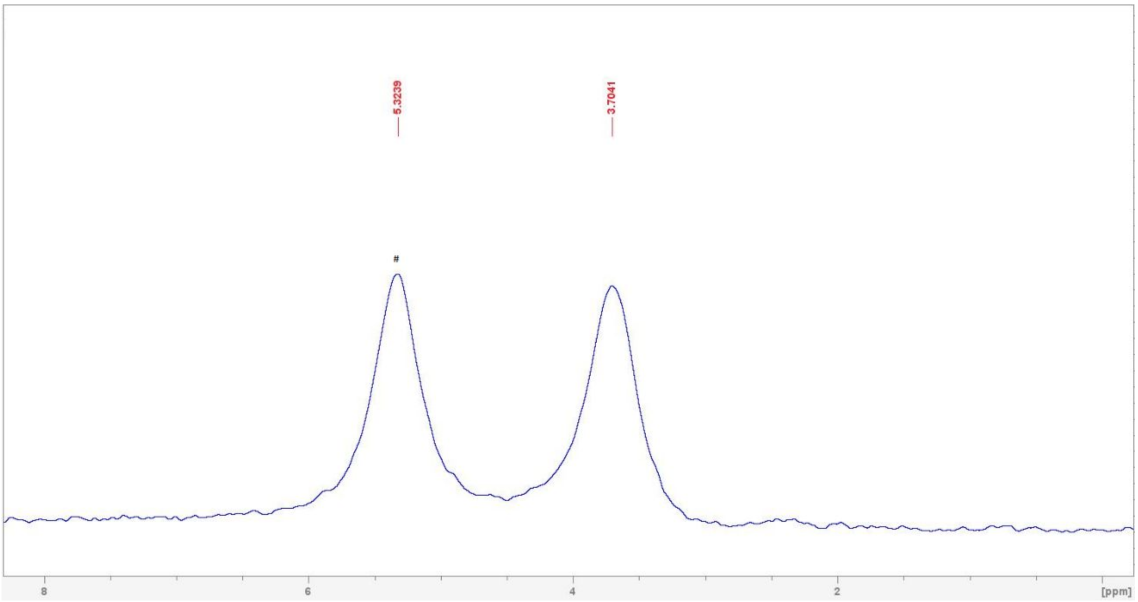

Figure S11. ^{2}H NMR spectrum (CH_2Cl_2, 60 MHz) of the H/D exchange in $^{n}Pr_3SiH$ (**Si-3**). (# denotes CD_2Cl_2 used as reference).

Ph_3SiD (Si-4)

Deuterium incorporation: 63%. Spectroscopic data for this product agree to those previously reported in the literature.[1,5]

^{1}H NMR (400 MHz, CD_2Cl_2): δ 7.61 (m, 6H, 6 H arom), 7.40 (m, 9H, 9 H arom), 5.51 (s, SiH).

^{2}H NMR (60 MHz, CH_2Cl_2): δ 5.77 (SiD).

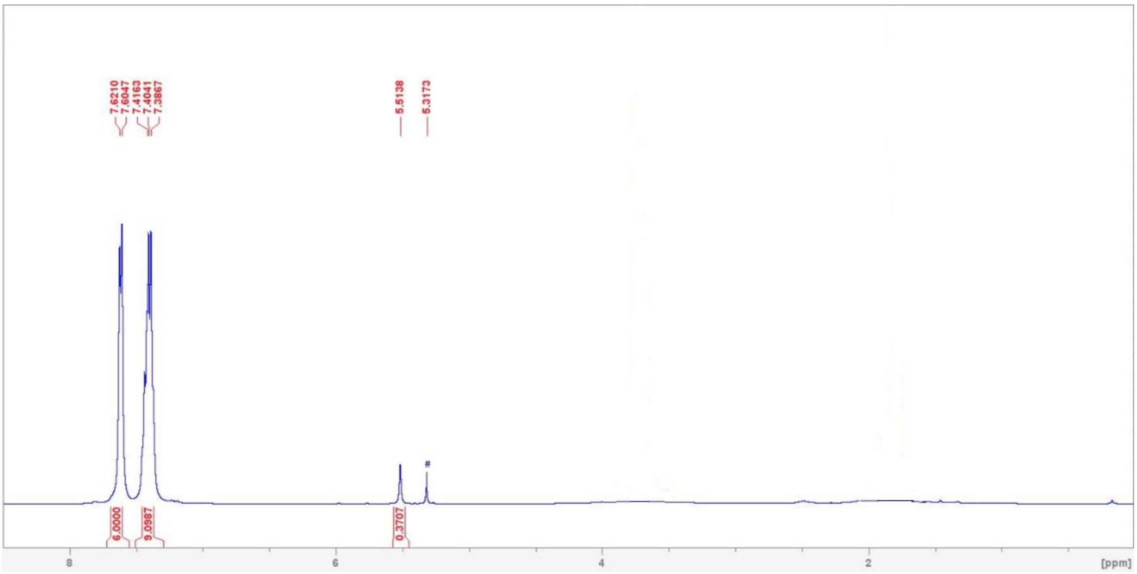

Figure S12. ^{1}H NMR spectrum (CD_2Cl_2, 400 MHz) of the H/D exchange in Ph_3SiH (**Si-4**). (# denotes residual CH_2Cl_2 from the deuterated solvent).

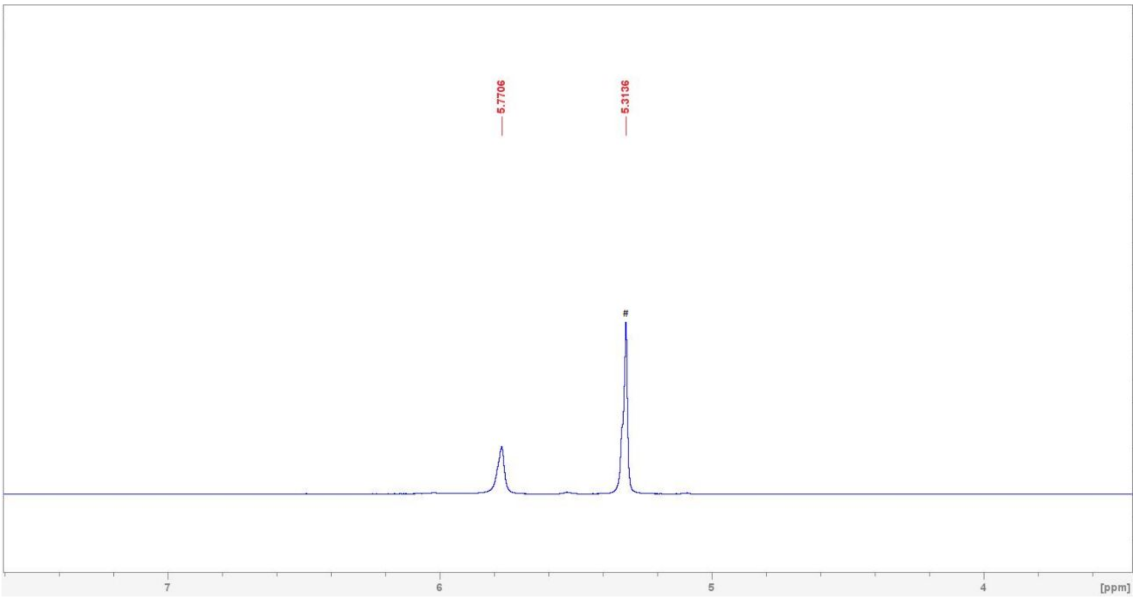

Figure S13. ^{2}H NMR spectrum (CH_2Cl_2, 60 MHz) of the H/D exchange in Ph_3SiH (**Si-4**). (# denotes CD_2Cl_2 used as reference).

1,2-(Me_2SiH)C_6H_4 (Si-5)

Deuterium incorporation: 80%. Spectroscopic data for this product agree to those previously reported in the literature.[6]

^{1}H NMR (400 MHz, CD_2Cl_2): δ 7.59 (m, 2H, 2 H arom), 7.36 (m, 2H, 2 H arom), 4.68 (hept, $^{3}J_{HH}$ = 3.6 Hz, SiH), 0.37 (s, 12H, 4CH_3).

^{2}H NMR (60 MHz, CH_2Cl_2): δ 4.85 (s, 1D, SiD).

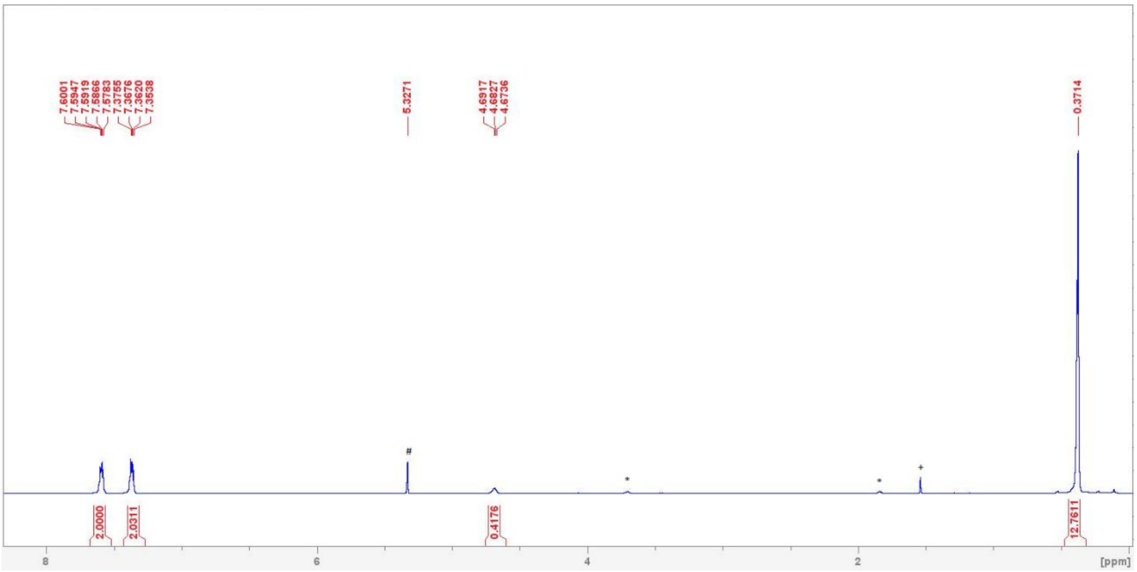

Figure S14. ^{1}H NMR spectrum (CD_2Cl_2, 400 MHz) of the H/D exchange in 1,2-(Me_2SiH)C_6H_4 (**Si-5**). (# denotes residual CH_2Cl_2 from the deuterated solvent, * denotes residual THF, + denotes H_2O).

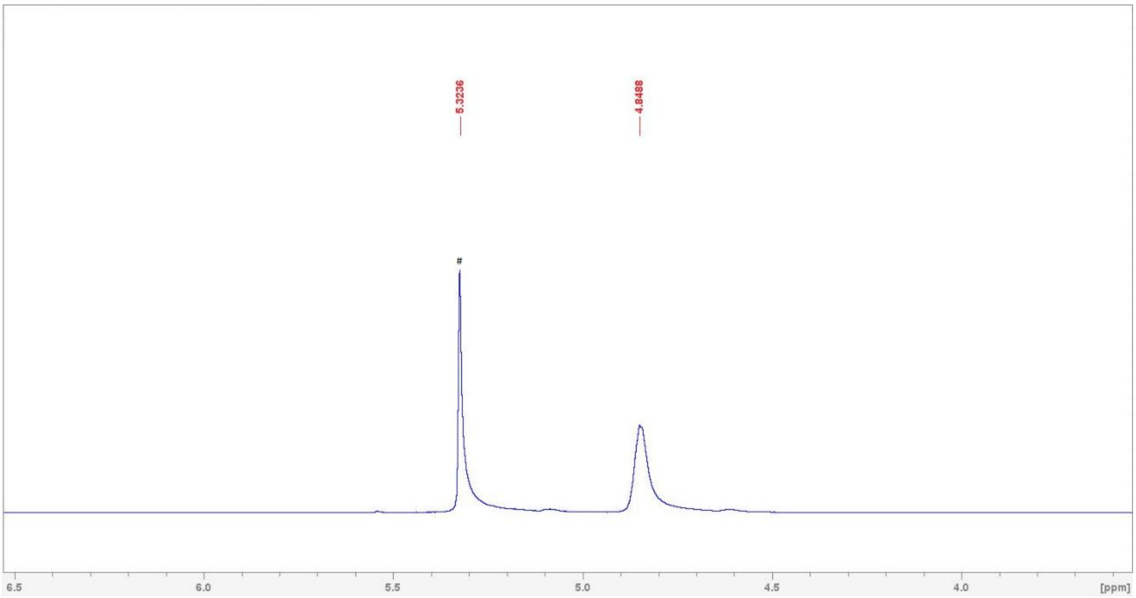

Figure S15. ^{2}H NMR spectrum (CH_2Cl_2, 60 MHz) of the H/D exchange in 1,2-(Me_2SiH)C_6H_4 (**Si-5**). (# denotes CD_2Cl_2 used as reference).

($(EtO)_3SiD$ (Si-6)

Deuterium incorporation: 93%. Spectroscopic data for this product agree to those previously reported in the literature.[1,7]

^{1}H NMR (400 MHz, $CDCl_3$): δ 4.34 (br s, SiH), 3.86 (m, $^{3}J_{HH}$ = 7.0 Hz, 6H, 3 CH_2), 1.24 (m, $^{3}J_{HH}$ = 7.0 Hz, 9H, 3 CH_3).

^{2}H NMR (60 MHz, $CHCl_3$): δ 4.38 (SiD).

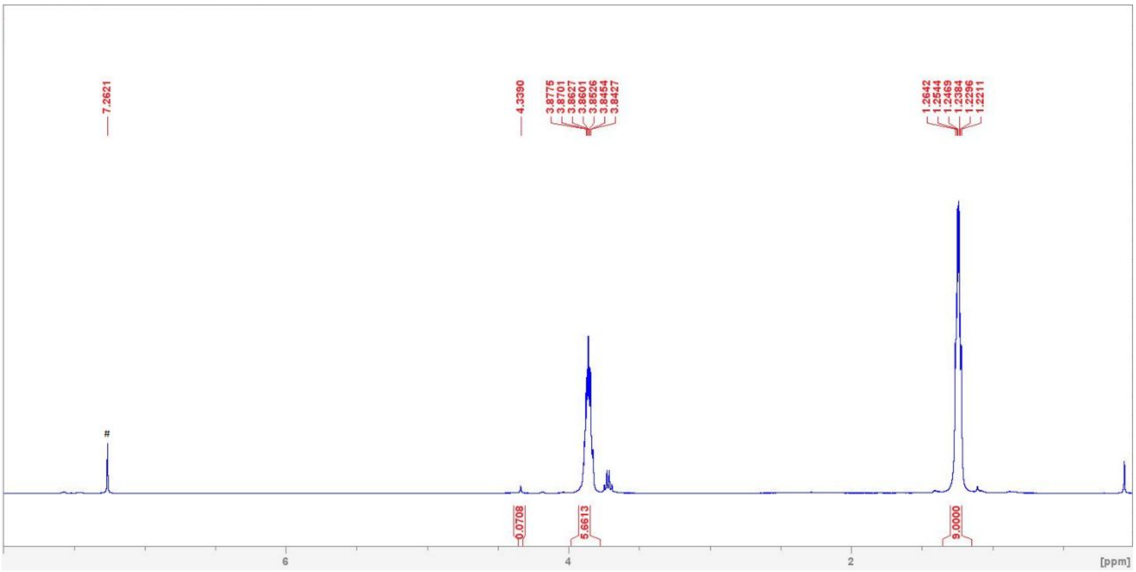

Figure S16. ^{1}H NMR spectrum ($CDCl_3$, 400 MHz) of the H/D exchange in $(EtO)_3SiH$ (**Si-6**). (# denotes residual $CHCl_3$ from the deuterated solvent).

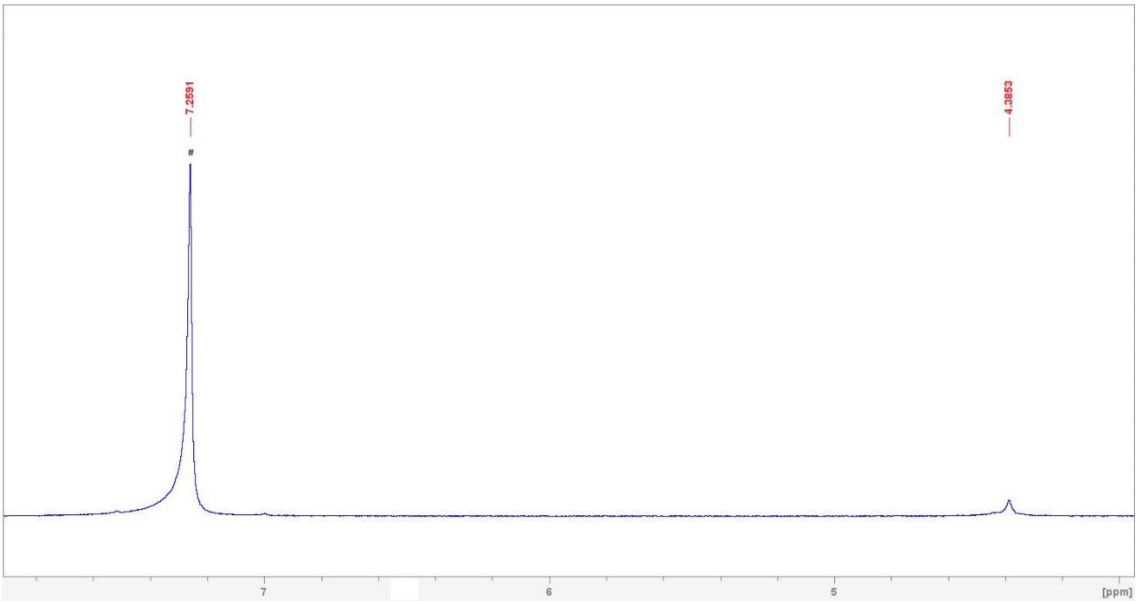

Figure S17. ^{2}H NMR spectrum ($CHCl_3$, 60 MHz) of the H/D exchange in $(EtO)_3SiH$ (**Si-6**). (# denotes $CDCl_3$ used as reference).

Et_3GeD (Ge-1)

Deuterium incorporation: 65%. Spectroscopic data for this product agree to those previously reported in the literature.[1,8]

^{1}H NMR (400 MHz, CD_2Cl_2): δ 3.64 (m, $^3J_{HH}$ = 3.0 Hz, SiH), 1.06 (t, $^3J_{HH}$ = 7.6 Hz, 9H, 3 CH_3), 0.82 (q, $^3J_{HH}$ = 7.6 Hz, 6H, 3 CH_2).

^{2}H NMR (60 MHz, CH_2Cl_2): δ 3.72 (GeD).

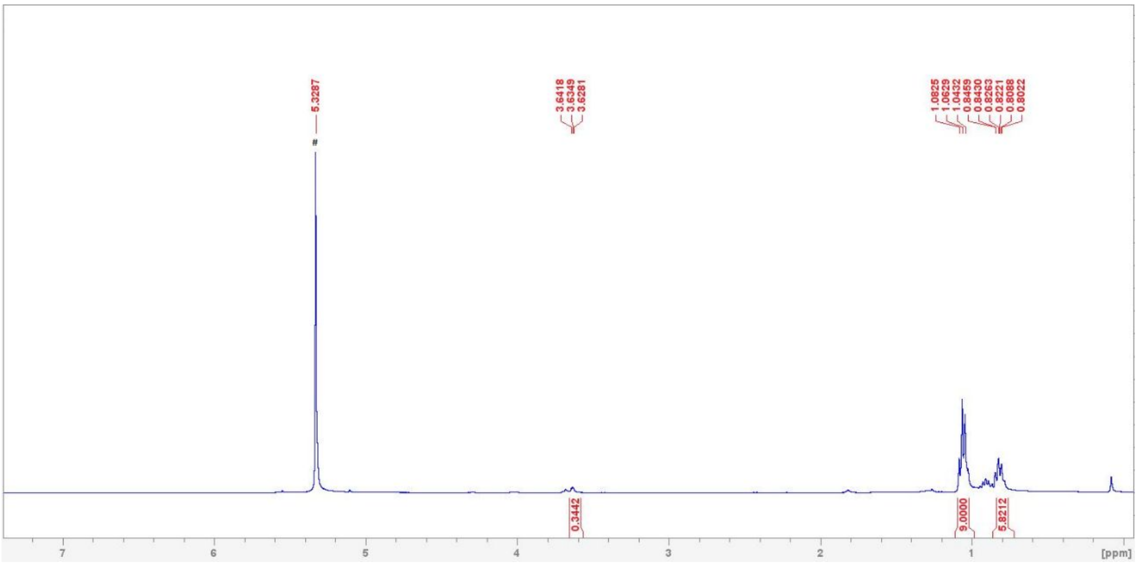

Figure S18. ^{1}H NMR spectrum (CD_2Cl_2, 400 MHz) of the H/D exchange in Et_3GeH (**Ge-1**). (# denotes residual CH_2Cl_2 from the deuterated solvent).

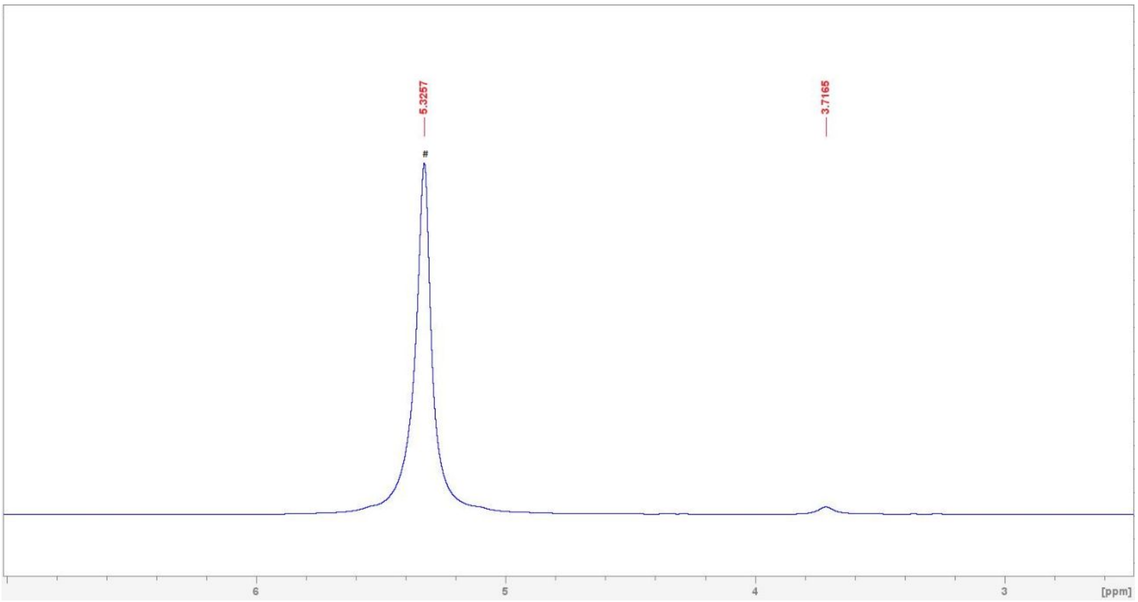

Figure S19. ^{2}H NMR spectrum (CH_2Cl_2, 60 MHz) of the H/D exchange in Et_3GeH (**Ge-1**). (# denotes CD_2Cl_2 used as reference).

$^{n}Bu_3SnD$ (Sn-1)

Deuterium incorporation: >99%. Spectroscopic data for this product agree to those previously reported in the literature.[1,9]

^{1}H NMR (400 MHz, CD_2Cl_2): δ 1.52 (m, 6H, 3 CH_2), 1.32 (m, 6H, 3 CH_2), 0.98 (m, $^{3}J_{HH}$ = 8.3 Hz, 6H, 3 CH_2), 0.89 (m, $^{3}J_{HH}$ = 7.5 Hz, 9H, 3 CH_3).

^{2}H NMR (60 MHz, CH_2Cl_2): δ 4.78 (SnD).

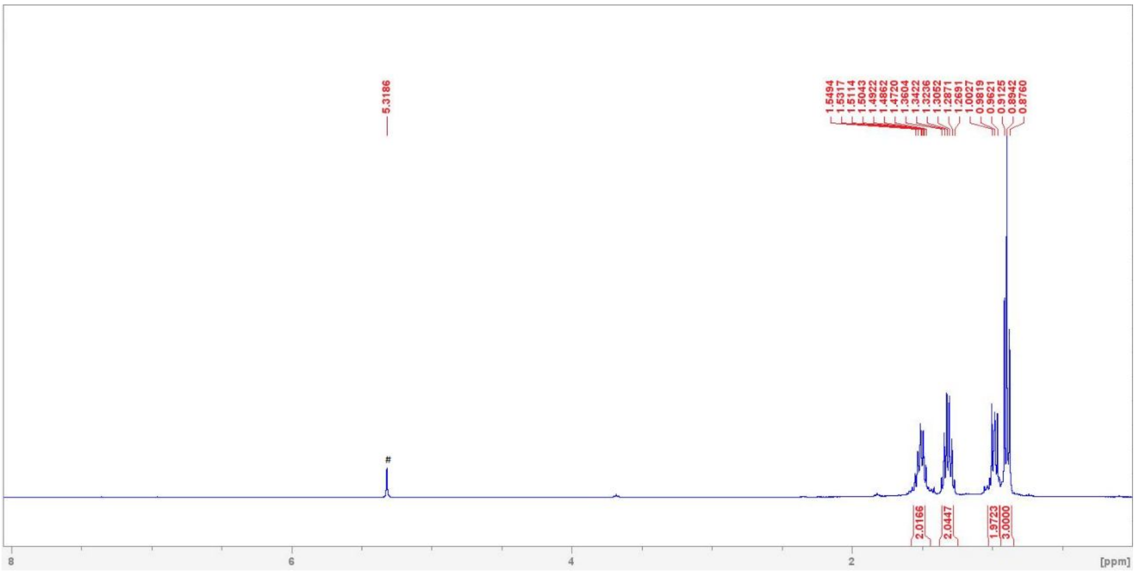

Figure S20. ^{1}H NMR spectrum (CD_2Cl_2, 400 MHz) of the H/D exchange in $^{n}Bu_3SnD$ (**Sn-1**). (# denotes residual CH_2Cl_2 from the deuterated solvent).

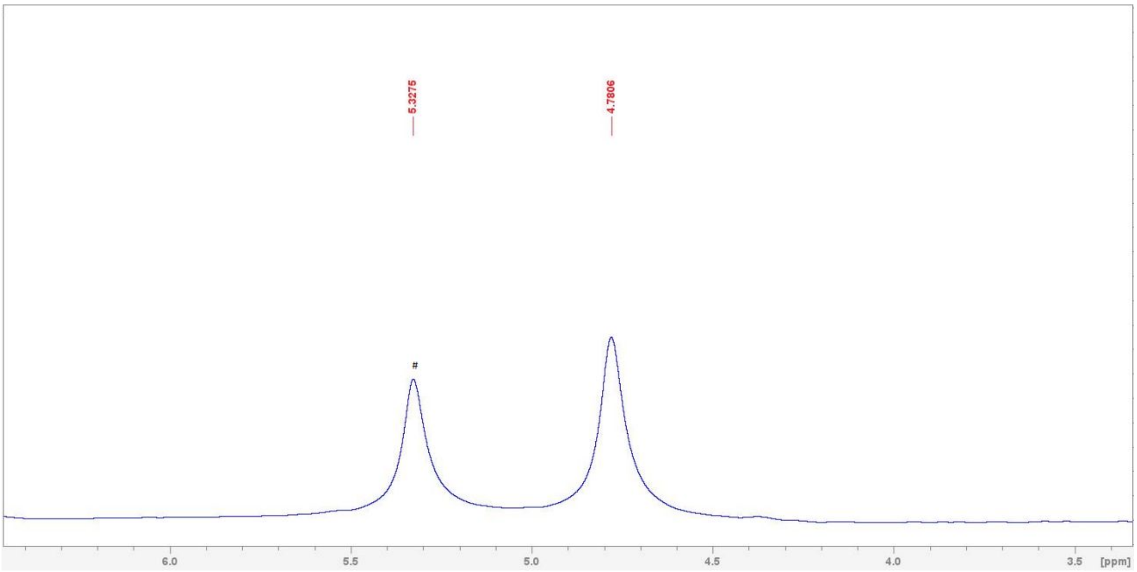

Figure S21. ^{2}H NMR spectrum (CH_2Cl_2, 60 MHz) of the H/D exchange in $^{n}Bu_3SnD$ (**Sn-1**). (# denotes CD_2Cl_2 used as reference).

S5. Additional catalytic experiments.

S5.1. Attempted deuteration of 2-phenylpyridine.

In a glovebox, a 25 mL Fisher-Porter vessel was charged with a solution of 2-phenylpyridine (55.36 µL, 0.38 mmol) and Ni·**MIC2**$^{0.2}$ (5.6 mg, 0.038 µmol) in THF (1.0 mL). The reactor was purged three times with D_2, and finally pressurized to 1 bar and heated to 55 ^{o}C. After 21 h, the reactor was slowly cooled down to room temperature and depressurized. An aliquot of the reaction mixture was filtered through a short pad of celite and brought to dryness. No H/D exchange was determined by ^{1}H and ^{2}H NMR spectroscopies.

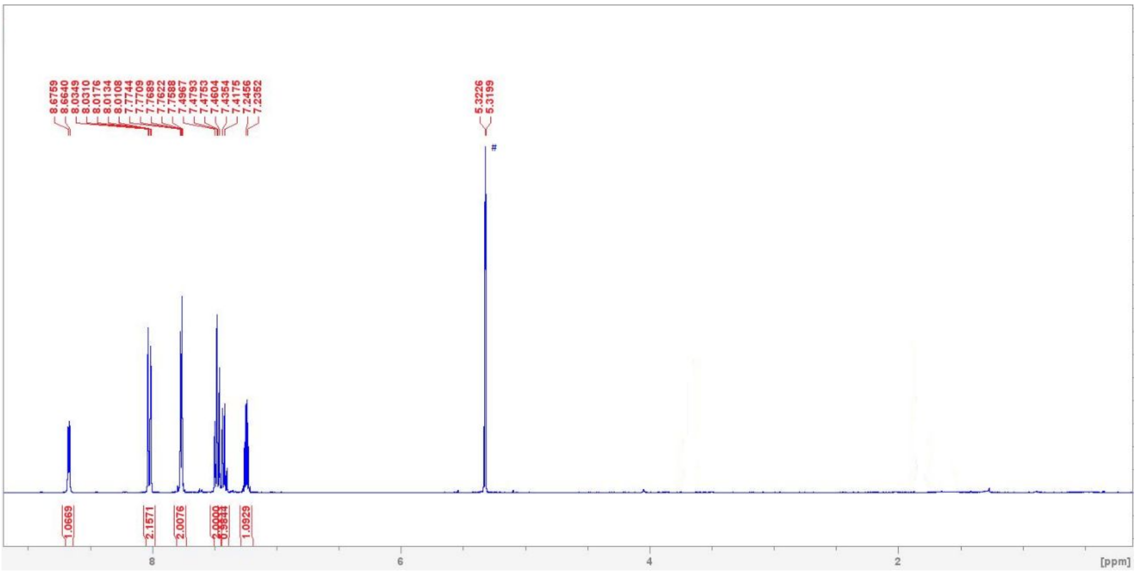

Figure S22. ^1H NMR spectrum (CD_2Cl_2, 400 MHz) of the attempted deuteration of 2-phenylpyridine. (# denotes residual CH_2Cl_2 from the deuterated solvent).

S5.2. Competition experiment.

Procedure for the selective H/D exchange of a 1:1 mixture of dimethylphenylsilane (Si-1) and 2-phenylpyridine. In a glovebox, a 25 mL Fisher-Porter vessel was charged with a solution of dimethylphenylsilane (**Si-1**) (83 µL, 0.68 mmol), 2-phenylpyridine (115 µL, 0.68 mmol) and Ni·**MIC1**$^{0.2}$ (2.0 mg, 13 µmol) in THF (1.0 mL). The reactor was purged three times with D_2, and finally pressurized to 1 bar and heated to 55 ^oC. After 21 h, the reactor was slowly cooled down to room temperature and depressurized. An aliquot of the reaction mixture was filtered through a short pad of celite and brought to dryness. The conversion and selectivity were determined by ^1H and ^2H NMR spectroscopies, respectively.

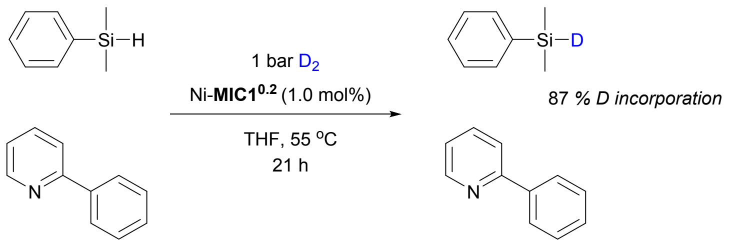

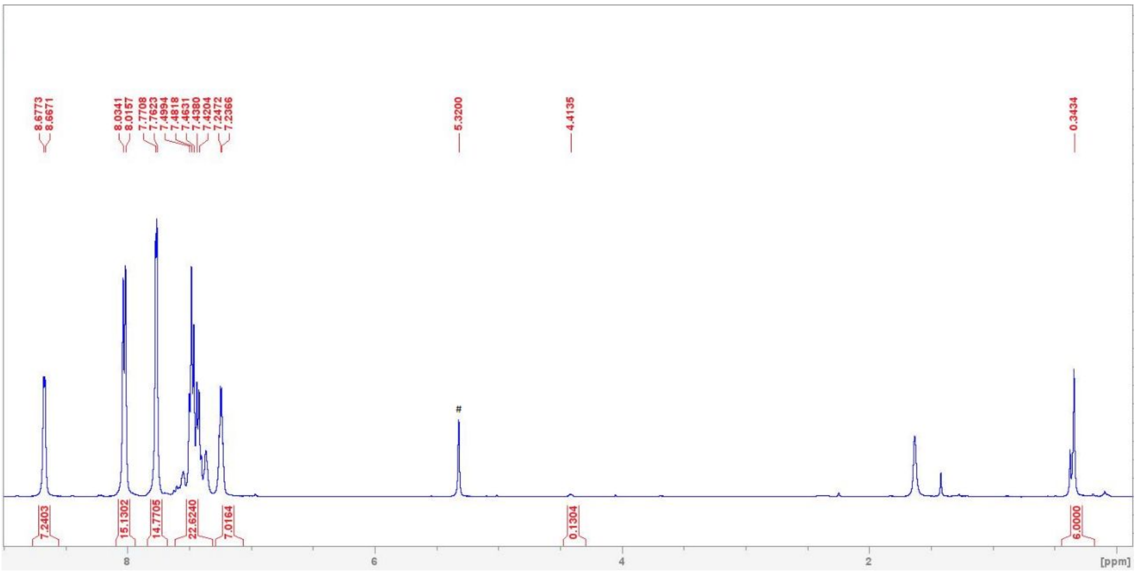

Figure S23. ^{1}H NMR spectrum (CD_2Cl_2, 400 MHz) of the H/D exchange in a 1:1 mixture of Me_2PhSiH (**Si-1**) and 2-phenylpyridine. (# denotes residual CH_2Cl_2 from the deuterated solvent).

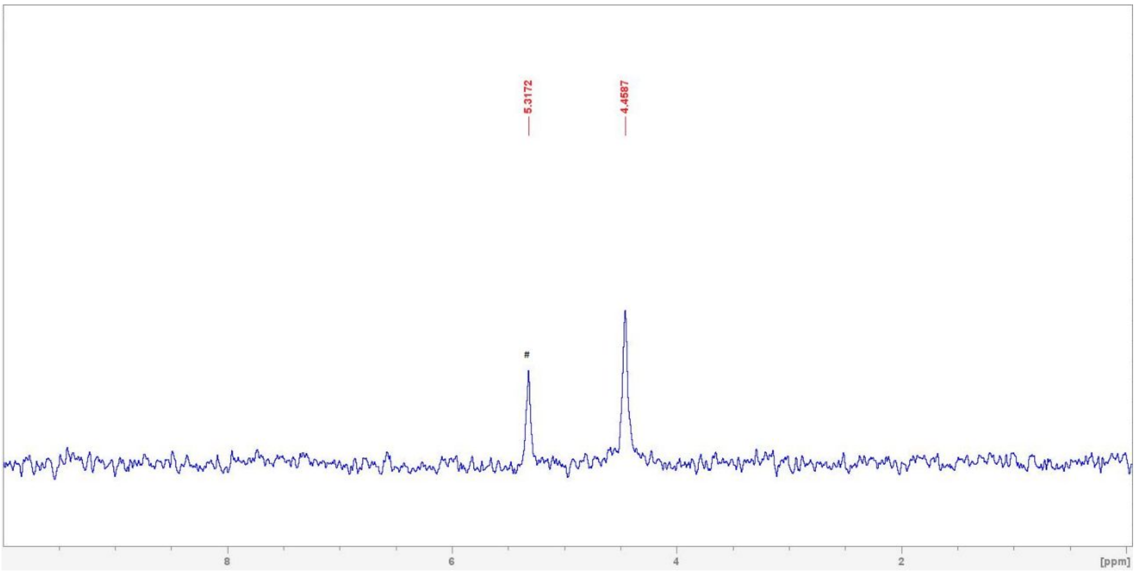

Figure S24. ^{2}H NMR spectrum (CH_2Cl_2, 60 MHz) of the H/D exchange in a 1:1 mixture of Me_2PhSiH (**Si-1**) and 2-phenylpyridine. (# denotes CD_2Cl_2 used as reference).

S5.3. Catalytic reaction using Ni ·MIC1$^{0.2}$ exposed to air.

In a glovebox, a 25 mL Fisher-Porter vessel was charged with a solution of dimethylphenylsilane (**Si-1**) (165 µL, 1.36 mmol), and Ni·**MIC1**$^{0.2}$ exposed to air for 5 days (2.0 mg) in THF (1.0 mL). The reactor was purged three times with D_2, and finally pressurized to 1 bar and heated to 55 ^oC. After 21 h, the reactor was slowly cooled down to room temperature and depressurized. An aliquot of the reaction mixture was filtered through a short pad of celite and brought to dryness.

The reaction was analyzed by ^{1}H NMR spectroscopy. ^{2}H NMR spectroscopy did not show any deuterated products.

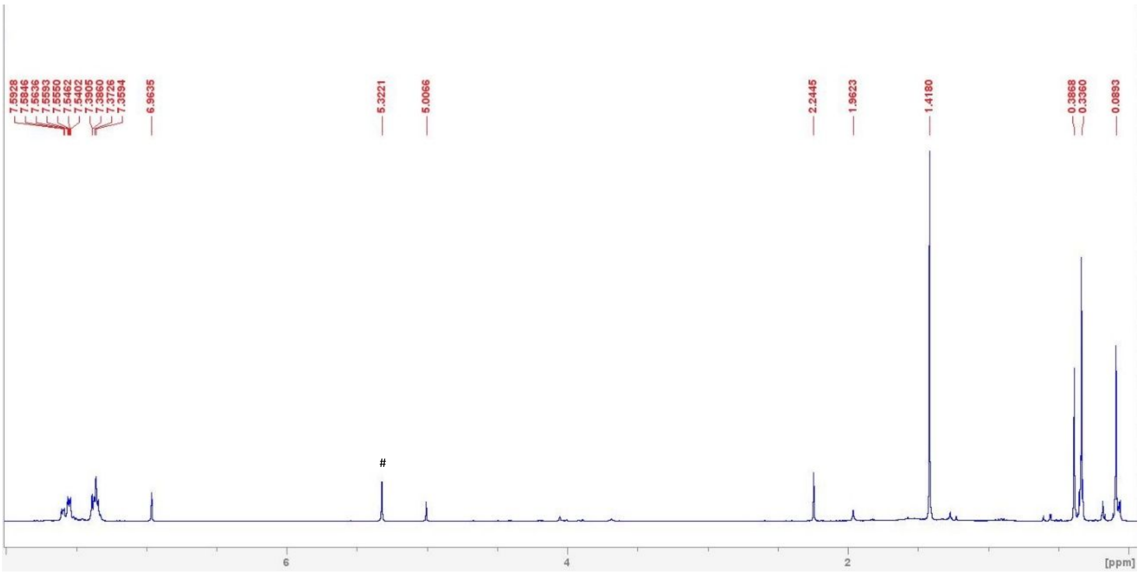

Figure S25. ^{1}H NMR spectrum (CD_2Cl_2, 400 MHz) of the reaction of Me_2PhSiH (**Si-1**) with D_2 using Ni·**MIC1**$^{0.2}$ exposed to air for 5 days. (# denotes residual CH_2Cl_2 from the deuterated solvent).

S6. References.

[1] Molinillo, P.; Puyo, M.; Vattier, F.; Lacroix, B.; Rendón, N.; Lara, P.; Suárez, A. Ruthenium nanoparticles stabilized by 1,2,3-triazolylidene ligands in the hydrogen isotope exchange of E–H bonds (E = B, Si, Ge, Sn) using deuterium gas. *Nanoscale* **2023**, *15* (35), 14488-14495.

[2] Wissing, M.; Studer, A. Tuning the Selectivity of AuPd Nanoalloys towards Selective Dehydrogenative Alkyne Silylation. *Chem. Eur. J.* **2019**, *25* (23), 5870-5874.

[3] Greb, L.; Tamke, S.; Paradies, J. Catalytic metal-free Si–N cross-dehydrocoupling. *Chem. Commun* **2014**, *50* (18), 2318-2320.

[4] Komuro, T.; Osawa, T.; Suzuki, R.; Mochizuki, D.; Higashi, H.; Tobita, H. Silyl–pyridine–amine pincer-ligated iridium complexes for catalytic silane deuteration via room temperature C–D bond activation of benzene-d6. *Chem. Commun.* **2019**, *55* (7), 957-960.

[5] Zhang, S.; Fallah, H.; Gardner, E. J.; Subrata, S.; Bertke, J. A.; Cundari, T. A Dinitrogen Dicopper(I) Complex via a Mixed-Valence Dicopper Hydride. *Angew. Chem. Int. Ed.* **2016**, *55* (34), 9927-9931.

[6] Campos, J.; Esqueda, A.; López-Serrano, J.; Sánchez, L.; Cossio, F. P.; de Cozar, A.; Álvarez, E.; Maya, C.; Carmona, E. A Cationic Rh(III) Complex That Efficiently Catalyzes Hydrogen Isotope Exchange in Hydrosilanes. *J. Am. Chem. Soc.* **2010**, *132* (47), 16765-16767.

[7] Smart, K.; Mothes-Martin, E.; Annaka, T.; Grellier, M. Silane Deuteration Catalyzed by Ruthenium Bis(dihydrogen) Complexes or Simple Metal Salts. *Adv. Synth. Catal.* **2014**, *356* (4), 759-764.

[8] Esteruelas, M. A.; Martínez, A.; Oliván, M.; Vélez, A. A General Rhodium Catalyst for the Deuteration of Boranes and Hydrides of the Group 14 Elements. *J. Org. Chem.* **2020**, *85* (23), 15693-15698.

[9] Lu, Y.; Yamago, S. One-Step Synthesis of Dendritic Highly Branched Polystyrenes by Organotellurium-Mediated Copolymerization of Styrene and a Dienyl Telluride Monomer. *Angew. Chem. Int. Ed.* **2019**, *58* (12), 3952-3956.
